# A novel reflective intelligence optimizer with machine learning (RIO-ML) for parameter estimation of photovoltaic models

**DOI:** 10.1038/s41598-026-60435-8

**Published:** 2026-07-09

**Authors:** Ahmed Bayoumi, Mahana M. Elbana, A. A. Nasef, M. E. Ali

**Affiliations:** Physics and Engineering Mathematics Department, Faculty of Engineering, Kafrelshiekh University, Kafrelshiekh, 33516 Egypt

**Keywords:** Photovoltaic, Parameter estimation, Machine learning, Metaheuristic optimization, Reflective intelligence, Adaptive control, Energy science and technology, Engineering, Mathematics and computing

## Abstract

This paper presents a new Reflective Intelligence Optimizer with Machine Learning (RIO-ML) approach to estimate the parameters of solar photovoltaic (PV) equivalent circuit models, which are highly nonlinear and multimodal, and hence require efficient handling by conventional optimizers. RIO-ML combines three major components: a multi-leader social learning algorithm with personal-best reflective memory, machine learning-driven adaptive control of important parameters using Multi-Layer Perceptron models, and progressive Gaussian refinement with reflective boundary treatment for improved convergence and robustness. The performance of RIO-ML is tested on the standard RTC France solar cell model with three different model settings: Single Diode Model (SDM) with 5 parameters, Double Diode Model (DDM) with 7 parameters, and Triple Diode Model (TDM) with 9 parameters, for 30 independent runs for each scenario. RIO-ML obtains the minimum RMSE of 8.739710 × 10^− 4^ A, 8.456760 × 10^− 4^, and 7.7546980 × 10^− 4^ A for SDM, DDM, and TDM models, respectively, with corresponding low mean RMSE values of 2.223109 × 10^− 3^ A, 2.282687 × 10^− 3^ A, and 1.717649 × 10^− 3^ A. Comparative studies reveal that RIO-ML performs better than some of the best metaheuristic algorithms available in the literature with respect to solution quality, convergence rate, and robustness, while maintaining the maximum absolute current errors less than 1.6 × 10^− 3^ A for all models. These findings clearly indicate that the developed RIO-ML approach is an effective and efficient tool for accurate estimation of PV parameter values.

## Introduction

 The rising global energy demand, accompanied by an urgent need to reduce greenhouse gas emissions and adapt to climate change, has catalyzed a worldwide transition toward renewable and resilient energy infrastructures^1^. Renewable energy is essential for fulfilling basic human needs and driving social and economic development, impacting everything from industrial revolutions to the modern quest for renewable resources. Since the world’s energy mix is currently undergoing a paradigm shift due to the urgent need to switch from fossil fuels to sustainable resources, renewable energy resources are now absolutely necessary in the effort to solve environmental problems and guarantee energy security^2,3^. With 240 GW built in a single year in 2022, the cumulative global installed capacity of PV technology surpassed 1 TW, contributing one-third of the total global renewable energy capacity^4^, demonstrating the exponential development rate. Solar energy, as an abundant resource that can be tapped without the generation of pollutants, is one of the most promising sources of renewable energy. The optimization of PV technology is therefore of utmost importance in the effort to make fewer uses of fossil fuels and lower carbon emissions, as the modeling of PV technology has been proven to have a direct effect on energy efficiency and sustainable development.

PV modeling is the basis for a number of critical applications such as maximum power point tracking (MPPT), analysis, and system optimization^5^. Accuracy of these models has a great influence on energy output forecasting, power conversion system efficiency, and economic viability of solar energy installation^6^. The accuracy of these models is directly related to the realism of energy output simulation, power conversion system efficiency, and economic feasibility of solar power installations. Among the different techniques used for modeling solar cells, the equivalent circuit models^7^, namely single diode, double diode, and triple diode models, have received widespread acceptance due to the optimal balance between the accuracy and complexity of the models^5,6^.

The issue of estimating parameters for PV models is inherently very difficult. There are many local optima, solutions are very dependent on the starting conditions, and the parameters are very linked to each other^8^. Traditional analytical methods are usually incapable of reflecting the nonlinear behavior of real PV cells under changing environmental conditions. Besides, classical optimization techniques are quite often trapped at a local minimum and thus they yield suboptimal solutions. The difficulty has stimulated a lot of research on metaheuristic optimization algorithms that could represent a good alternative for this nonlinear and multimodal optimization problem.

Although the domain has witnessed remarkable development, current metaheuristic methods still encounter several challenges. A number of algorithms undergo premature convergence when applied to the high, dimensional search spaces of triple diode models. Commonly used algorithms like Particle Swarm Optimization (PSO)^9^, Genetic Algorithm (GA)^10^, Differential Evolution(DE)^11^, Grey Wolf Optimizer(GWO)^12^, Harris hawks optimization (HHO)^13^, Grasshopper Optimization Algorithm(GOA)^14^, Electric Eel Foraging Optimizer (EEFO)^15^, Prairie Dog Optimizer(PDO)^16^, Whale Optimization Algorithm (WOA)^17^, Crayfish Optimization Algorithm^18^, Phantom Based Search Optimizer (PhaBSO)^19^, Zebra Optimization Algorithm (ZOA)^20^, Dandelion Optimizer (DO)^21^, and Artificial Hummingbird Algorithm (AHA)^22^ lack a good exploration, exploitation balance and thereby either converge very slowly or get stuck in local optima.

Recent research has demonstrated significant progress through novel metaheuristic approaches. The improved Artificial Protozoa Optimizer (iAPO) achieved RMSE values of 9.8602E-04 for single diode models^23^, while the Puma Optimizer with Lambert W-function (POLam) reached 7.218852E-04 for RTC France cells^24^. The Marine Predators Algorithm (MPA) obtained 7.561E-04 for triple diode models^25^, and the Chaotic Gorilla Troops Optimizer (CGTO) explored alternative circuit configurations using analytical solutions^26^. Improved Tasmanian Devil Optimization (ITDO) has been developed to enhance the performance of the original TDO, including improvements to the exploitation phase, increasing the frequency of prey detection and attacks in the target area^27^, Comparative studies on multijunction solar cells evaluated multiple algorithms (GWO, AHA, TSA, WSA, MFO) across different model complexities^28^, Augmented Mountain Gazelle Optimizer based on the Improved Berndt–Hall–Hall–Hausman method (AMGO_IB3H_), for enhancing the convergence behavior and exploration–exploitation balance of the original MGO algorithm^29^, while the Puma Optimizer Algorithm (POA)^30^, the Arctic Puffin Optimization (APO) algorithm^31^, and the Starfish Optimization Algorithm (SFOA)^32^ showed effectiveness in solar cells^33^ and transformer parameter estimation^34^ respectively. However, systematic integration of machine learning-based adaptive mechanisms with metaheuristic optimization remains underexplored in PV parameter estimation. What is more, nearly all the parameter estimation techniques that have been proposed so far treat it as an optimization problem only and thus miss out on the benefits that domain, specific knowledge and adaptive learning^35,36^ techniques offer in paving the way for better solutions and faster convergence.

More recently, hybrid approaches have tried to solve the problems of the previous methods by using a mixture of optimization techniques or adding machine learning methods^37–39^. Nevertheless, the methods sometimes do not have systematic frameworks for adaptive strategy selection and parameter tuning during the optimization process. The use of reflective intelligence mechanisms^40^, i.e., when an algorithm learns from its own search behavior and makes dynamic adjustments to its strategies, is still an new area in PV parameter estimation.

This research presents the Reflective Intelligence Optimizer with Machine Learning (RIO-ML), a novel approach to tackle these challenges through a hybrid architecture that synergistically combines metaheuristic optimization with machine learning-based adaptive control^41^. The proposed RIO-ML algorithm integrates three main innovations: (1) a reflective intelligence mechanism that balances social learning from multiple leader solutions with personal best memory, enabling effective exploration-exploitation trade-off; (2) machine learning-driven parameter adaptation, where Multi-Layer Perceptron (MLP)^42^ regression models dynamically predict optimal parameter values based on real-time optimization characteristics including search progress, population diversity, and fitness improvement rate; and (3) a progressive convergence strategy that gradually shifts from exploration to exploitation through adaptive Gaussian refinement^43^ with linearly increasing refinement probability over iterations.

The RIO-ML framework’s effectiveness is thoroughly tested with real, world data from a well, known RTC France solar cell, a benchmark dataset widely used in the PV industry for validating parameter estimation algorithms. Extensive testing is done on three levels of model complexity: single diode model (SDM) with five parameters, double diode model (DDM) with seven parameters, and triple diode model (TDM) with nine parameters^44–48^. This step, by, step evaluation gives a comprehensive review of the algorithm’s scalability and performance stability across different problem sizes.

*The main contributions of this study are as follows*

1. Algorithmic Innovation: development of RIO-ML, a revolutionary hybrid optimization approach that overcomes the limitations of existing metaheuristic methods by integrating machine learning and reflective intelligence processes for better parameter estimation performance.
2. Comprehensive Model Coverage: systematic treatment for SDM, DDM, and TDM models, showing outstanding accuracy and providing valuable information on algorithmic performance for different levels of model complexity.
3. Rigorous Validation: comprehensive statistical analysis of 30 independent trials, encompassing convergence characteristics, solution accuracy, and robustness assessment, and rigorous experimental testing using RTC France solar cell data.
4. Practical Applicability: Excellent accuracy and convergence performance are introduced, which improves the modeling of the PV system. The smallest RMSE of the SDM model, DDM model, and TDM model is 8.739710 × 10^− 4^ A, 8.456760 × 10^− 4^, and 7.7546980 × 10^− 4^ A, respectively.
5. The RIO-ML algorithm is compared with five well-known optimization algorithms (ZOA, DO, PhaBSO, WOA, and GWO) across three PV models (SDM, DDM, and TDM), demonstrating superior accuracy with the lowest RMSE in all cases.

The rest of the paper is organized as follows. Section 2 describes single-, double- and triple-diode models and presents the problem formulations and models. Section 3 introduces the proposed RIO-ML method and the adaptive machine learning method in it. Details of the experiments, results and analysis are given in Sect. 4. Finally, Sect. 5 concludes the paper and indicates future work.

## Problem definition

### Single diode dodel (SDM)

**Fig. 1:**
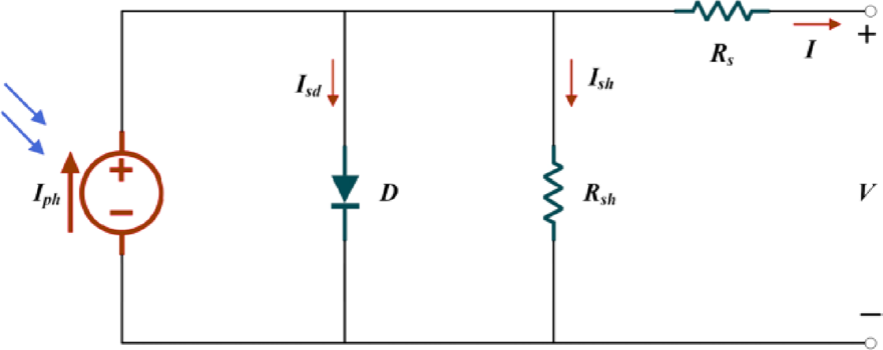
SDM diagram.

The equivalent circuit of SDM is shown in Fig. 1. In this model, the output voltage an current are denoted by V and I, respectively. The output current of the SDM mathematically expressed in Eq. (1).

1 $$\:I={I}_{ph}-{I}_{sh}-{I}_{sd}={I}_{ph}-\frac{V+I{R}_{s}}{{R}_{sh}}-{I}_{ssd}\left[exp\left(\frac{q\left(V+I{R}_{s}\right)}{nkT}\right)-1\right]$$

In this equation, I_ph_ represents the photocurrent, while I_sh_, I_sd_, and I_ssd_ correspond to the shunt current, diode current, and diode saturation current, respectively. The terms R_s_, and R_sh_ denote the series and shunt resistances, respectively. The parameter n is the diode ideality factor, whereas T, k, and q denote the absolute temperature, Boltzmann constant, and the electron charge, respectively. To accurate modeling for SMD requires estimating the values of I_ph_, I_ssd_, n, R_s_, and R_sh_.

### Double diode model (DDM)

**Fig. 2:**
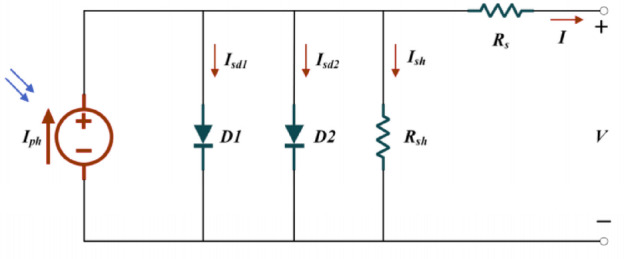
DDM digram.

The equivalent circuit of DDM is shown in Fig. 2, the electrical expression for the output current I is expressed in Eq. (2).

2 $$\:\begin{array}{c}I={I}_{ph}-{I}_{sh}-{I}_{sd1}-{I}_{sd2}\\\:{I}_{ph}-\frac{V+I{R}_{s}}{{R}_{sh}}-{I}_{sd1}\left[exp\left(\frac{q\left(V+I{R}_{s}\right)}{{n}_{1}kT}\right)-1\right]-{I}_{sd2}\left[exp\left(\frac{q\left(V+I{R}_{s}\right)}{{n}_{2}kT}\right)-1\right]\end{array}$$

where I_sd1_ and I_sd2_ denote the first and second diode currents, respectively, I_ssd1_ and I_ssd2_ denote the corresponding diode saturation currents, and n_1_ and n_2_ mean the corresponding ideal factors. The values of I_ph_, I_ssd1_, I_ssd2_, n_1_, n_2_, R_s_, and R_sh_ must be estimated for an accurate model.

### Triple diode model (TDM)

**Fig. 3:**
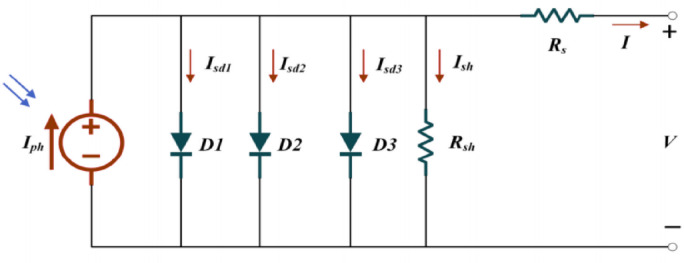
TDM diagram.

The equivalent diagram of TDM is shown in Fig. 3, the electrical expression for the output current I is expressed in Eq. (3).

3 $$\:I={I}_{ph}-{I}_{sh}-{\sum\:}_{j=1}^{3}{I}_{sdj}={I}_{ph}-\frac{V+I{R}_{s}}{{R}_{sh}}-{\sum\:}_{j=1}^{3}{I}_{ssdj}\left[exp\left(\frac{q\left(V+I{R}_{s}\right)}{{n}_{j}kT}\right)-1\right]$$

where *I*_*sdj*_, *I*_*ssdj*_, and nj represent j the diode current, the saturation current, and the ideal factor, respectively. The values of *I*_*ph*_, *I*_*ssd1*_, *I*_*ssd2*_, *I*_*ssd3*_, *n*_*1*_, *n*_*2*_, *n*_*3*_, *R*_*s*_, *R*_*sh*_ must be estimated for an accurate model.

### Problem formulation

**Fig. 4:**
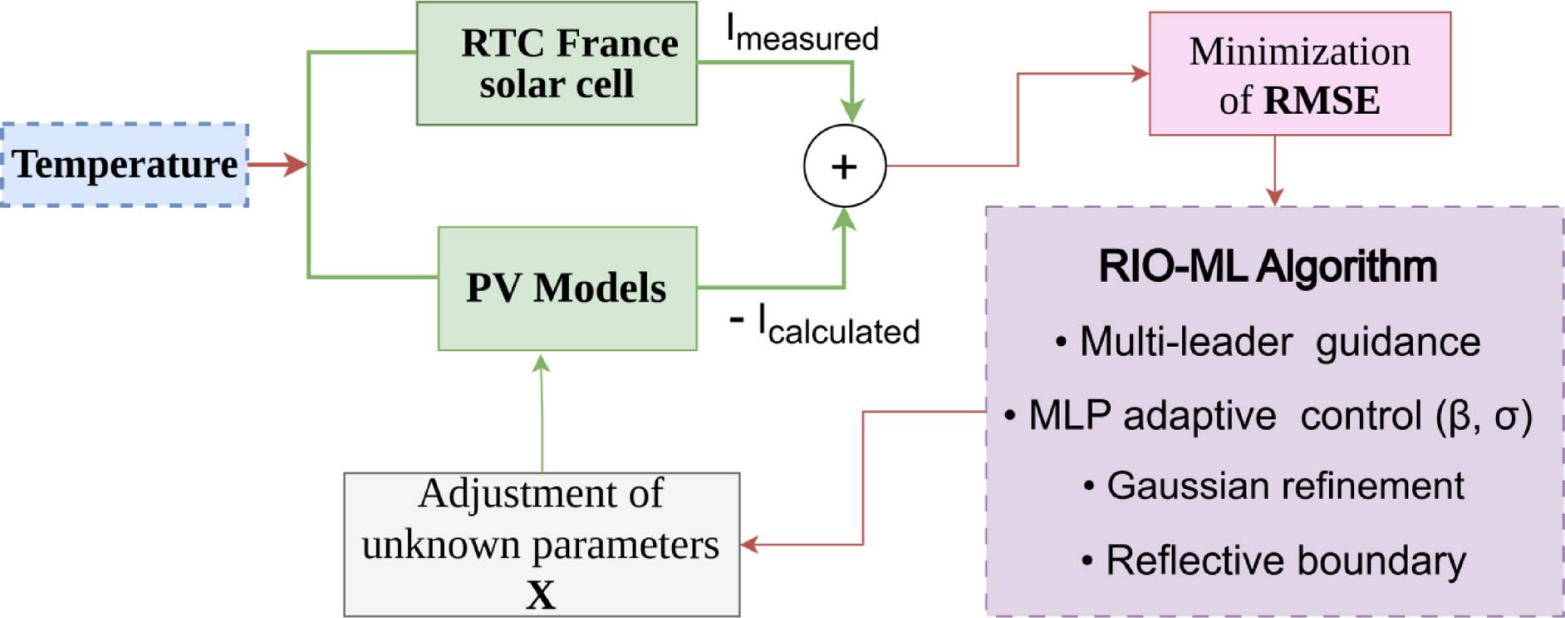
Overview for parameters estimation of PV Cell models using RIO-ML.

Figure 4 provides an overview of the solar module parameter identification process. As depicted, the output voltage and current, along with solar Temperature (33 °c), are utilized as essential inputs to the PV cell model. These models are the most typical ones. In order for an optimization method to determine exact parameter values, the equations are converted into related optimization difficulties. The Objective Function (OF) shows the least difference among the estimated and experimental data when the ideal parameter values are used. The majority of algorithms, employ the total RMSE as their assessment criterion. The RMSE may also be used to assess the disparity between predicted and actual results. So, RMSE is nominated as the OF as shown in Eq. (4)

4 $$\:OF\left(X\right)=RMSE\left(X\right)=\sqrt{\frac{1}{M}{\sum\:}_{d=1}^{M}f{\left({V}_{L},{I}_{L},X\right)}^{2}}$$

where X stands for the vector holding the to be estimated unknown parameters and M is the total number of experimental I-V data points. the function f_M_(V_L_,I_L_,X) represents the error between the estimated current (I_est_) and the current measured (I_t_). Specifically, for the single diode model, the function f_SM_(V_L_,I_L_,X) is derived by subtracting Eq. (1) from the measured current value (I_t_), as shown in Eq. (5). Similarly, Eq. (6) were obtained by using Eq. (2), and (7) respectively, instead of Eq. (3).

5 $$\:\left\{\begin{array}{c}{f}_{SDM}\left({V}_{L},{I}_{L},X\right)={I}_{ph}-{I}_{sd}\left[exp\left(\frac{q\left({V}_{L}+{R}_{s}{I}_{L}\right)}{nkT}\right)-1\right]-\frac{{V}_{L}+{R}_{s}{I}_{L}}{{R}_{sh}}-{I}_{L}\\\:X=\left\{{I}_{ph},{I}_{sd},{R}_{s},{R}_{sh},n\right\}\end{array}\right.$$

6 $$\:\left\{\begin{array}{c}{f}_{DDM}\left({V}_{L},{I}_{L},X\right)={I}_{ph}-{I}_{sd1}\left[exp\left(\frac{q\left({V}_{L}+{R}_{s}{I}_{L}\right)}{{n}_{1}kT}\right)-1\right]-{I}_{sd2}\left[exp\left(\frac{q\left({V}_{L}+{R}_{s}{I}_{L}\right)}{{n}_{2}kT}\right)-1\right]\\\:-\frac{{V}_{L}+{R}_{s}{I}_{L}}{{R}_{sh}}-{I}_{L}\\\:X=\left\{{I}_{ph},{I}_{sd1},{I}_{sd2},{R}_{s},{R}_{sh},{n}_{1},{n}_{2}\right\}\end{array}\right.$$

7 $$\:\left\{\begin{array}{c}{f}_{TDM}\left(V,I,X\right)={I}_{ph}-{I}_{sd1}\left[exp\left(\frac{V+I{R}_{s}}{{n}_{1}{V}_{t}}\right)-1\right]\\\:-{I}_{sd2}\left[exp\left(\frac{V+I{R}_{s}}{{n}_{2}{V}_{t}}\right)-1\right]-{I}_{sd3}\left[exp\left(\frac{V+I{R}_{s}}{{n}_{3}{V}_{t}}\right)-1\right]-\frac{V+I{R}_{s}}{{R}_{sh}}-I\\\:X=\left\{{I}_{ph},{I}_{sd1},{I}_{sd2},{I}_{sd3},{R}_{s},{R}_{sh},{n}_{1},{n}_{2},{n}_{3}\right\}\end{array}\right.$$

### Parameter setting and benchmark dataset

In this study, the PV parameter extraction problem is formulated and validated using the RTC France benchmark I–V data measured under standard test conditions of 303.15 K and 1000 W/m². Accordingly, the irradiance effect is implicitly reflected in the experimental data used for parameter estimation. The present formulation is therefore intended for benchmark evaluation under fixed operating conditions.

The PV cell and module specifications, are provided in Table 1, and the experimental I–V data of the R.T.C (26 data points)^49^. France solar cell are presented in Table 2.

To assess the RIO-ML algorithm and ensure a fair comparison with other Algorithms, namely namely GWO, WOA, ZOA, DO, and PhaBSO, the general simulation environment, optimization settings, and algorithm-specific control parameters, including initialization strategies and method-dependent coefficients for all compared algorithms are listed in Table 3.

**Table 1:** Specifications of commercial R.T.C solar cell.

Operation temperature (K)	303.15
Operation radiation (W/m²)	1000
Maximum power (Pmpp) (W)	0.3106
Voltage at MPP (Vmpp) (V)	0.4518
Current at MPP (Impp) (A)	0.6874
Open circuit voltage (Voc) (V)	0.5727
Short circuit current (Isc) (A)	0.7605
FF (fill factor)	0.7130 (71.30%)

**Table 2:** Experimental I-V data for R.T.C. france solar cell (at 33 °C).

Index	Experimental voltage (V)	Experimental current (A)
0	−0.2057	0.764000
1	−0.1291	0.762000
2	−0.0588	0.760500
3	0.0057	0.760500
4	0.0646	0.760000
5	0.1185	0.759000
6	0.1678	0.757000
7	0.2132	0.757000
8	0.2545	0.755500
9	0.2924	0.754000
10	0.3269	0.750500
11	0.3585	0.746500
12	0.3873	0.738500
13	0.4137	0.728000
14	0.4373	0.706500
15	0.4590	0.675500
16	0.4784	0.632000
17	0.4960	0.573000
18	0.5119	0.499000
19	0.5265	0.413000
20	0.5398	0.316500
21	0.5521	0.212000
22	0.5633	0.103500
23	0.5736	−0.010000
24	0.5833	−0.123000
25	0.5900	−0.210000

**Table 3:** Simulation environment, optimization algorithm settings, and algorithm-specific control parameters for all algorithms.

Paramaters	GWO	WOA	ZOA	DO	PhaBSO	RIO-ML (proposed)
General settings						
Independent run time	30					
Population size	30					
Number of iteration	500					
Processor	Intel^®^ Core™ i7-7500U CPU @ 2.70 GHz					
Programming environment	Python 3.11.6					
Operating system	Linux					
Initialization	Random uniform	Random uniform	Random uniform	Random uniform	Latin Hypercube	Random uniform
Algorithm-specific control parameters						
a (linearly decreases)	2 → 0	2 → 0				2 → 0
a₂ (linearly decreases)		−1 → −2				
b (spiral constant)		1				
Lion escape factor R			0.1			
Lévy flight exponent β				1.5		
α parameter				Adaptive (α = rand × (t²/T² − 2t/T + 1))		
Phantom energy update					+ 0.15/−0.03	
Stagnation detection					20 iters, tol = 1 × 10⁻⁸	
β range (social weight)						0.2 → 0.9
σ range (Gaussian step)						0.5 → 0.001
MLP hidden layers						(5, 5)
ML update frequency						Every 10 iterations (after iter. 20)

## RIO-ML algorithm

The RIO-ML (Reflexive Intelligence Optimizer with Machine Learning) is a hybrid metaheuristic optimization algorithm that combines adaptive machine learning for dynamic parameter adjustment with principles of whale optimization algorithms. It leverages both individual reflective learning based on each agent’s personal best location and multi-leader guidance with the three best responses. The convergence speed and accuracy for the optimized solutions are ensured by two MLP regression models that dynamically adjust critical control parameters based on real-time optimization feedback during the search process.

### Overview of proposed method

A novel hybrid metaheuristic method (RIO-ML), incorporates principles of whale optimization, reflective intelligence, and adaptive machine learning techniques into the optimization process. The algorithm maintains a population of search agents that develop through a synergistic combination of social learning and reflective intelligence. Each agent in the population is guided by three leader positions (L₁, L₂, L₃) representing the best solution, the second-best solution, and the third-best solution discovered thus far, and the agent’s own personal best position. A dual-memory system is used to handle global exploration through social interaction and local exploitation through individual experience.

The core innovation of the RIO-ML method uses its machine learning system to adjust control parameters automatically through its Multi-Layer Perceptron regression models, which determine essential control setting values during the optimization process. Two MLP neural networks, which have three-input neuron layers and two hidden neuron layers with ReLU activation and one output neuron layer, continuously learn the optimal values for:

1. The Social-Reflective Balance function (β) controls the balance between social learning from leaders and personal reflective learning from best positions, which is bounded within the range of 0.2 to 0.9.
2. Gaussian Refinement (σ) Controls the intensity of local search refinement around promising solutions, bounded within [0.001, 0.5].

These ML models are trained online using historical optimization data comprising three key features—progress ratio $\:t/{T}_{max}$, population diversity (measured as mean standard deviation across all dimensions), and fitness improvement magnitude (absolute change in best fitness between consecutive iterations). The first 20 iterations of the process establish baseline results through mathematical decay parameters, which apply to $\:\beta\:$ and to $\:\sigma\:$ through their respective $\:{\beta\:}_{max}$ to $\:{\beta\:}_{min}$and $\:{\sigma\:}_{init}$ to $\:{\sigma\:}_{final}$ decay functions. The system retrains its machine learning models every 10 iterations after the 20th iteration to forecast adaptive parameter values which match the present characteristics of the search landscape. In case ML training fails or insufficient data is available, the algorithm automatically falls back to the linear/exponential decay schedules. This adaptive technique allows the method to dynamically shift between exploration and exploitation phases based on learned patterns rather than predetermined schedules.

The position update mechanism uses a three-part design which operates through multiple stages to implement its system. First, a weighted social vector is constructed by aggregating information from the three leader positions through whale optimization coefficients. For each leader $\:k\in\:\left\{1,2,3\right\}$, coefficients $\:{A}_{k}=2{a}_{r}-a$ and $\:{C}_{k}=2r$ (where r are random values in [0,1]) determine the distance $\:{D}_{k}=\left|{C}_{k}\times\:{L}_{k}-{x}_{i}\right|$ and candidate position $\:{X}_{k}={L}_{k}-{A}_{k}\times\:{D}_{k}$. The social vector combines these influences with weights emphasizing higher-quality solutions: $Social = \left( {2X_{1} + 1.5X_{2} + X_{3} } \right)/4.5$. Second, this social component is hybridized with the agent’s reflective vector (personal best) using the learned $\:\beta\:$ coefficient: $\:\overrightarrow{{x}_{new}}=\beta\:\times\:\mathrm{Social}+\left(1-\beta\:\right)\times\:\overrightarrow{{pbest}_{i}}$, creating a balance between collective intelligence and individual memory. Third, a Gaussian refinement phase applies probabilistic perturbations with intensity σ to solutions: $\:{x}_{new}={x}_{new}+0.5\times\:G\times\:\left({L}_{1}-{x}_{new}\right)$, where $\:G\sim\:N\left(0,\sigma\:\right)$, facilitating fine-grained local search in promising regions. The refinement probability (p_refine) increases linearly from 0.3 to 1.0 over iterations, gradually intensifying exploitation as the search progresses. The convergence parameter a is decreased linearly from 2 to 0 to control the exploration-exploitation transition. The method uses a boundary handling mechanism which reflects agents back into the search area when they exceed the established boundaries to maintain solution feasibility and increase solution diversity rather than simply clamped: $\:\mathrm{if\:}{x}_{j}>{ub}_{j}\mathrm{\:then\:}{x}_{j}={ub}_{j}-\left|{x}_{j}-{ub}_{j}\right|$, and $\:\mathrm{if\:}{x}_{j}<{lb}_{j}\mathrm{\:then\:}{x}_{j}={lb}_{j}+\left|{x}_{j}-{lb}_{j}\right|,$ preserving population variance while ensuring constraint satisfaction. The integration of machine learning, whale-inspired multi-leader guidance, and adaptive refinement enables RIO-ML to effectively navigate complex, multimodal optimization landscapes characteristic of PV parameter estimation problems.

### RIO-ML mathematical model

The mathematical formulation of RIO-ML is organized into distinct operational phases, which use separate equations to manage different aspects of the optimization process.

#### The key phases of RIO-ML

##### Initialization phase

Through the initialization phase, the basic population structure gets created because N agents get randomly placed throughout the D-dimensional search space between the limits of the [lb, ub] boundary. The initial position of each agent serves as its personal best ($\:{pbest}_{i}$) until improved through subsequent iterations. The population assesses the complete group of solutions to determine three leading solutions, which serve as L₁, L₂, and L₃ to direct social learning during optimization. The initialization phase includes:

1. Agent Initialization:

For each agent i ∈ {1, 2, …, N} and each dimension j ∈ {1, 2, …, D} as shown in Eq. (8).

8 $$\:{x}_{ij}={lb}_{j}+rand\left(0,1\right)\times\:\left({ub}_{j}-{lb}_{j}\right)$$

(2) Personal Best Initialization:

Initialize personal best positions and scores as shown in Eqs. (9 and 10).

9 $$\:{pbest}_{i}={x}_{i}$$

10 $$\:{\left({pbest}_{score}\right)}_{i}=f\left({x}_{i}\right)$$

(3) Leader Identification:

Identify three best solutions (leaders) from the population as shown in Eqs. (11,12 and 13).

11 $$\:{L}_{1}=argminf\left({x}_{i}\right)$$

12 $$\:{L}_{2}=arg{min}_{{x}_{i}\notin\:{L}_{1}}f\left({x}_{i}\right)$$

13 $$\:{L}_{3}={argmin}_{i\ne\:{L}_{1},{L}_{2}}f\left({x}_{i}\right)$$

##### Main optimization loop

The main optimization loop describes the core iterative process where agents develop their positions through adaptive learning and intelligent search strategies. This phase combines multiple approaches that use real-time diversity monitoring and machine learning to adjust parameters and social guidance based on whale optimization and reflective intelligence for individual learning. Each iteration combines exploration through multi-leader guidance with exploitation through Gaussian refinement, while the ML models use their continuous learning process to create the best parameter settings, which they derive from observing the patterns of search activities. The main optimization loop encompasses:

1. Progress and Diversity Metrics:

Search progress (normalized iteration) as shown in Eq. (14).

14 $$\:progress=t/{T}_{max}$$

Population diversity (average standard deviation across dimensions) as shown in Eq. (15).

15 $$diversity = \left( {1/D} \right)\sum\nolimits_{{j = 1}}^{D} {std\left( {x._{j} } \right)}$$

Fitness improvement (absolute change in best score) as shown in Eq. (16).

16 $$\:improvement=\left|\left(f{\left({L}_{1}\right)}^{t}-f{\left({L}_{1}\right)}^{t-1}\right)\right|$$

(2) Machine Learning Adaptation:

Feature vector for ML models as shown in Eq. (17).

17 $$X{\text{ }} = {\text{ }}\left[ {progress,{\text{ }}diversity,{\text{ }}improvement} \right]$$

MLP regression for β (social-reflective balance) as shown in Eqs. (18 and 19).

18 $$\:{\beta\:}^{t}=MLP\left(\beta\:\right)\left({X}^{t}\right)$$

19 $$\:{\beta\:}^{t}\in\:\left[{\beta\:}_{min},{\beta\:}_{max}\right]=\left[0.2,0.9\right]$$

MLP regression for σ (Gaussian refinement) as shown in Eqs. (20 and 21).

20 $$\:{\sigma\:}^{t}=MLP\left(\sigma\:\right)\left({X}^{t}\right)$$

21 $$\:{\sigma\:}^{t}\in\:\left[{\sigma\:}_{final},{\sigma\:}_{init}\right]=\left[0.001,0.5\right]$$

MLP Architecture (for both models) includes :

Input layer: 3 neurons (progress, diversity, improvement), Hidden layer 1: 5 neurons with ReLU activation, Hidden layer 2: 5 neurons with ReLU activation and Output layer: 1 neuron (predicted parameter value).

Training Strategy includes :

- Historical data :
  22 $$\:{X}_{hist}=\left[{X}^{1},{X}^{2},...,{X}^{t}\right]$$
- *Target values*:
  23 $$Y_{{\beta \:_{{hist}} }} = \left[ {\beta ^{1} ,\beta ^{2} ,...,\beta ^{t} } \right],\:Y_{{\sigma \:_{{hist}} }} = \left[ {\sigma ^{1} ,\sigma ^{2} ,...,\sigma ^{t} } \right]$$
- Training frequency: Every 10 iterations after iteration 20.
- Fallback: Linear decay if training fails or insufficient data.

(3) Fallback Linear Decay (Before ML Stabilizes)

Beta linear decay as shown in Eq. (24).

24 $$\:{\beta\:}^{t}={\beta\:}_{max}-\left({\beta\:}_{max}-{\beta\:}_{min}\right)\times\:progress$$

Sigma exponential decay *as shown in* Eq. (25).

25 $$\:{\sigma\:}^{t}={\sigma\:}_{init}\times\:{\left({\sigma\:}_{final}/{\sigma\:}_{init}\right)}^{progress}$$

(4) Whale Optimization Components

Convergence parameter (decreases linearly from 2 to 0) as shown in Eq. (26).

26 $$\:{a}^{t}=2-2\times\:progress$$

Refinement probability (increases from 0.3 to 1.0) as shown in Eq. (27).

27 $$\:{p}_{refine}=0.3+\left(0.7\times\:progress\right)$$

(5) Social Vector Calculation

For each dimension j, calculate influence from three leaders:

Leader 1 influence as shown in Eqs. (28, 29, 30 and 31).

28 $$\:{A}_{1}=2\times\:a\times\:{r}_{1}-a$$

29 $$\:{C}_{1}=2\times\:{r}_{2}$$

30 $$\:{D}_{1}=\left|\left({C}_{1}\times\:{L}_{1j}-{x}_{ij}\right)\right|$$

31 $$\:{X}_{1}={L}_{1j}-{A}_{1}\times\:{D}_{1}$$

Leader 2 influence as shown in Eqs. (32, 33, 34 and 35).

32 $$\:{A}_{2}=2\times\:a\times\:{r}_{3}-a$$

33 $$\:{C}_{2}=2\times\:{r}_{4}$$

34 $$\:{D}_{2}=\left|\left({C}_{2}\times\:{L}_{2j}-{x}_{ij}\right)\right|$$

35 $$\:{X}_{2}={L}_{2j}-{A}_{2}\times\:{D}_{2}$$

Leader 3 influence as shown in Eqs. (36, 37, 38 and 39).

36 $$\:{A}_{3}=2\times\:a\times\:{r}_{5}-a$$

37 $$\:{C}_{3}=2\times\:{r}_{6}$$

38 $$\:{D}_{3}=\left|\left({C}_{3}\times\:{L}_{3j}-{x}_{ij}\right)\right|$$

39 $$\:{X}_{3}={L}_{3j}-{A}_{3}\times\:{D}_{3}$$

Weighted combination of leader influences as shown in Eq. (40).

40 $$\:{Social}_{j}=\left(2\times\:{X}_{1}+1.5\times\:{X}_{2}+{X}_{3}\right)/4.5$$

*Where* $\:{r}_{1},{r}_{2},{r}_{3},{r}_{4},{r}_{5},{r}_{6}\sim\:U\left(0,1\right)$ are independent random numbers.

(6) Position Update with Reflective Intelligence

Combine social learning with reflective learning as shown in Eq. (41).

41 $$\:{x}_{i}^{t+1}=\beta\:\times\:{Social}_{i}+\left(1-\beta\:\right)\times\:{pbest}_{i}$$

(7) Gaussian Refinement

With probability prefine, apply Gaussian perturbation as shown in Eqs. (42 and 43).

42 $$\:G\sim\:N\left(0,\sigma\:\right)$$

43 $$\:{x}_{i}^{t+1}={x}_{i}^{t+1}+0.5\times\:G\times\:\left({L}_{1}-{x}_{i}^{t+1}\right)$$

(8) Reflective Boundary Handling

Ensure solutions remain within bounds using reflection as shown in Eqs. (44 and 45).

44 $$\:If{x}_{ij}>{ub}_{j}:{x}_{ij}={ub}_{j}-\left|\left({x}_{ij}-{ub}_{j}\right)\right|$$

45 $$\:If{x}_{ij}<{lb}_{j}:{x}_{ij}={lb}_{j}+\left|\left({x}_{ij}-{lb}_{j}\right)\right|$$

(9) Personal Best Update

Update personal best if current solution is better as shown in Eq. (46).

46 $$\:Iff\left({x}_{i}^{t+1}\right)<f\left({pbest}_{i}\right):{pbest}_{i}={x}_{i}^{t+1}$$

#### Key features of RIO-ML

1. Adaptive Parameter Control: Machine learning models use Adaptive Parameter Control to adjust both dynamically β and σ according to current search conditions, allowing the algorithm to automatically manage its exploration and exploitation phases.
2. Multi-Leader Guidance: The method applies weighted contributions from the three best solutions, which include the global best solution with a weight of 2.0, the second-best solution with a weight of 1.5, and the third-best solution with a weight of 1.0.
3. Reflective Intelligence: Each agent maintains and learns from its personal best position, combining global knowledge with individual experience.
4. Progressive Refinement: Adaptive Gaussian perturbations allow for precise adjustment of solutions in subsequent iterations.
5. Reflective Boundary Handling: Solutions exceeding bounds are returned to the search space, maintaining diversity.
6. Learning-Based Adaptation: MLP models learn from optimization history to predict optimal parameter values, improving performance over time.

#### Gaussian refinement and ML integration

Conventional metaheuristic algorithms use control parameters that stay fixed or just decay in a straight line for the whole search, so they kind of get stuck and can’t really adapt as the search landscape keeps changing. With RIO-ML instead, it uses two MLP regressors that keep an eye on three search state indicators, and then they dynamically tune the social weight β, which lives in [0.2, 0.9], along with the Gaussian perturbation step σ in the range [0.001, 0.5] based on what’s happening during the run. This mechanism provides three concrete benefits:

1. Search behavior: In early iterations, when population diversity is high and improvement is rapid, the ML regressors maintain larger σ and higher exploration weight, promoting broad search. As convergence progresses and diversity decreases, the regressors shift toward smaller σ and stronger exploitation, enabling fine-grained local refinement around promising regions a capability that fixed-parameter methods cannot replicate.
2. Convergence quality: The adaptive transition between exploration and exploitation prevents premature stagnation.
3. Solution accuracy: By adjusting the step size and social influence to what is really going on in the search at every stage, RIO-ML delivers strictly lower final RMSE numbers for all three solar cell models (SDM, DDM, and TDM).

### Pseudo-code and flowchart of the proposed RIO-ML algorithm

**Fig. 5:**
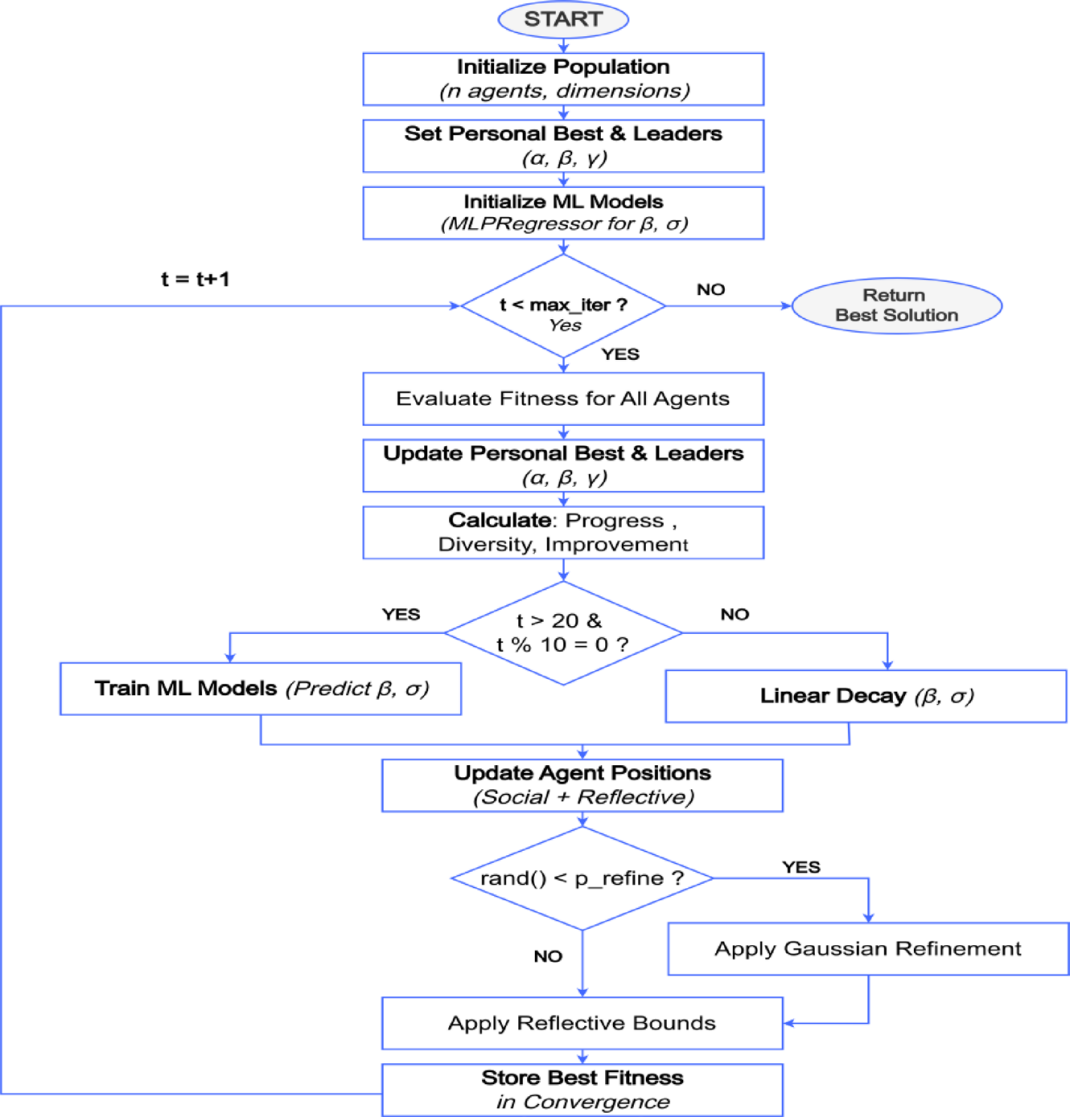
Flowchart of the proposed RIO-ML algorithm.

The RIO-ML workflow from its starting point to its final step is shown in Algorithm 1 which Table 4 demonstrates. Figure 5 displays the flowchart which shows the ML-based parameter adaptation starting from iteration 20 and the Gaussian refinement that $\:{p}_{refine}$ controls and the system’s reflective boundary processing.

**Algorithm 1:**
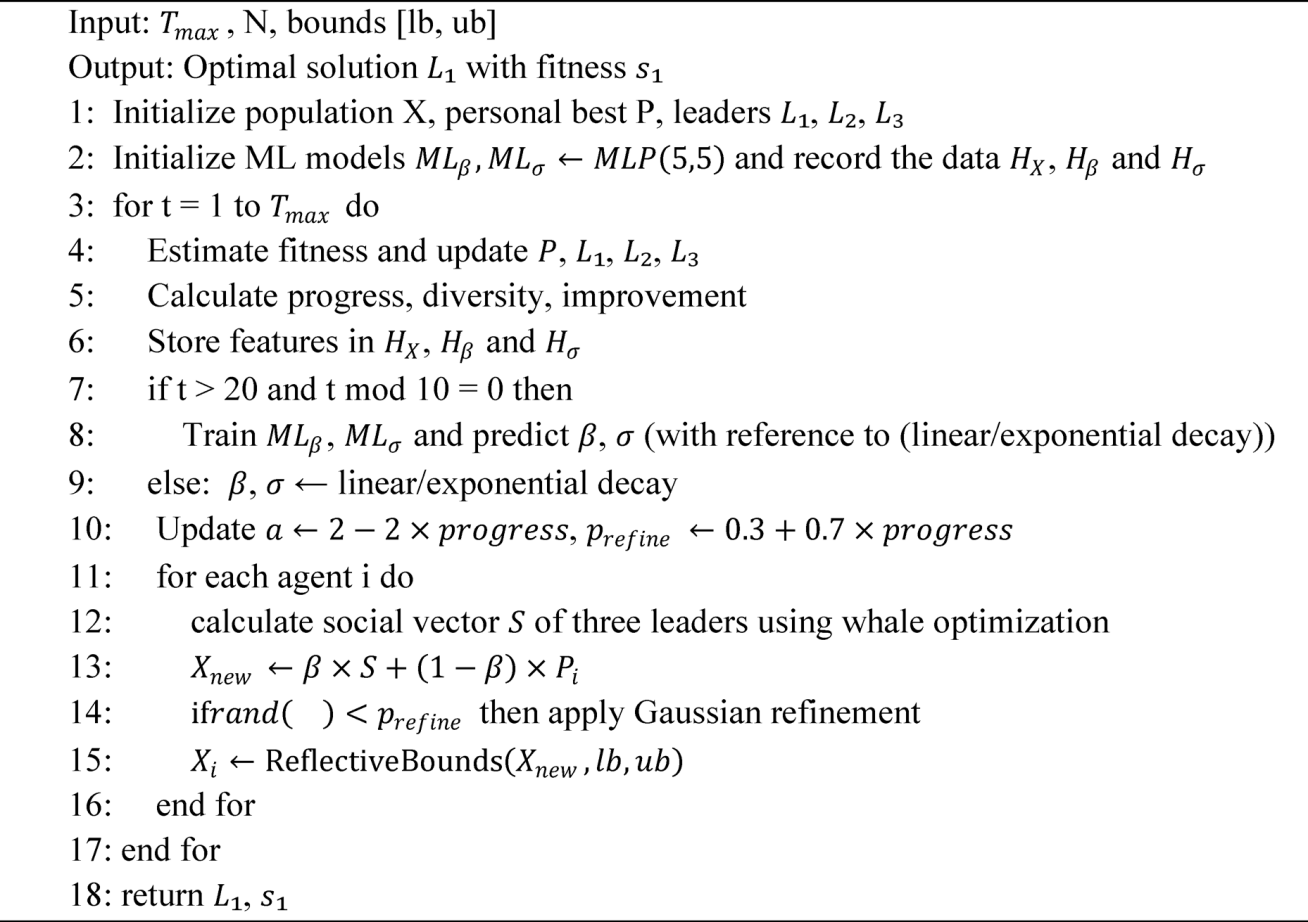
Reflective intelligence optimizer with machine learning (RIO-ML).

**Table 4:** Key features the proposed RIO-ML algorithm.

Line	Description
Line 1–2:	Initialization with ML models and personal best tracking
Line 4:	Fitness evaluation and three-leader selection mechanism
Line 5–6:	Population diversity and convergence monitoring
Line 7–9:	Adaptive ML-based parameter tuning (β, σ)
Line 10:	Update control parameters (a,$\:{p}_{refine}$)
Line 12:	Weighted multi-leader social learning
Line 13:	Hybrid social-reflective position construction
Line 14:	Stochastic Gaussian refinement
Line 15:	Reflective boundary handling mechanism
Complexity	$\:O\left(\left({T}_{max}\times\:N\times\:\mathrm{dim}\right)+\left({ML}_{train}\right)\right)$

## Results and discussion

To evaluate the performance of the RIO-ML algorithm, it was tested against five benchmark optimization algorithms, like ZOA, DO, PhaBSO, WOA, and GWO. Each algorithm was independently executed for 30 runs, with 500 iterations per run to ensure a statistically reliable comparison. The performance evaluation was conducted using both statistical analysis and convergence behavior for RMSE. The RMSE statistics which included best, worst, mean, Median, Variance and standard deviation values served as robustness measurement while convergence curves to study how well algorithms handled exploration and exploitation during their iterative process.

### Single diode model results

The RIO-ML algorithm was first evaluated on the Single Diode Model (SDM) with five parameters. The algorithm demonstrated exceptional performance with a minimum RMSE of 8.739710e-04 A achieved in Run 30, as shown in Table 8, which represents the best solution found across all 30 independent runs. The RMSE results statistical analysis for RIO-ML and other algorithms are presented in Table 5, The results demonstrate the lower RMSE values of the proposed RIO-ML approach. In addition, the standard deviation of RMSE for RIO-ML is 1.423158e − 03. which reflects the robustness of the algorithm.

The optimal parameters of SDM, identified by the best run for RIO-ML and other algorithms are presented in Table 6.

**Table 5:** Statistical metrics of RIO-ML, ZOA, DO, PhaBSO, WOA, and GWO for SDM.

RMSE	Optimization algorithms					
	RIO-ML	ZOA	DO	PhaBSO	WOA	GWO
Min	8.739710e-04	1.1582255e-03	1.3352770e-03	9.084225e-04	1.368912e-03	1.2917508e-03
Mean	2.223109e-03	2.6418240e-03	2.3591237e-03	1.878476e-03	1.069160e-02	8.2883753e-03
Max	7.984237e-03	1.0151689e-02	4.9139428e-03	2.972931e-03	4.111775e-02	4.5135225e-02
Median	1.876745e-03	1.9367503e-03	2.3790427e-03	2.037314e-03	6.732761e-03	3.5476857e-03
Std	1.423158e-03	2.0300199e-03	7.434558e-04	5.272433e-04	1.021813e-02	1.2619906e-02
Variance	2.025378e-06	4.1209807e-06	5.5272649e-07	2.779855e-07	1.044101e-04	1.5926204e-04

**Table 6:** Optimal parameters of RIO-ML, ZOA, DO, PhaBSO, WOA, and GWO for SDM.

Parameter	LB	UB	Optimal parameters of optimization algorithms					
			RIO-ML	ZOA	DO	PhaBSO	WOA	GWO
Iph (A)	0	1	0.760406	0.76068123	0.76139377	0.760512	0.759319	0.76108983
Isd (A)	0	1e-6	3.147993e-07	4.3984e-07	4.6278e-07	3.883486e-07	4.127568e-07	4.7478e-07
Rs (Ω)	0	0.5	0.036700	0.03521404	0.03471670	0.035842	0.035721	0.03495136
Rsh (Ω)	0	100	56.446444	64.55778002	52.85399709	61.452208	68.535849	63.81536124
n	1	5	1.478446	1.51289884	1.51850215	1.499970	1.506478	1.52093931

Table 7 shows the experimental versus predicted current values for all 26 data points using the best run parameters. The maximum absolute error is only 1.595761e − 03 A, occurring at index 12, while the mean absolute error is 7.46e − 04 A. These low error values demonstrate excellent agreement between the model predictions and experimental measurements (Table 8).

**Table 7:** Experimental vs. predicted current of RIO-ML for SDMl (Best run).

Index	Voltage (V)	I_exp (A)	I_pred (A)	Error (A)	Error %
0	−0.2057	0.764000	0.763554	4.464044e-04	0.0584
1	−0.1291	0.762000	0.762197	−1.974186e-04	0.0259
2	−0.0588	0.760500	0.760953	−4.526805e-04	0.0595
3	0.0057	0.760500	0.759810	6.898548e-04	0.0907
4	0.0646	0.760000	0.758765	1.235265e-03	0.1625
5	0.1185	0.759000	0.757800	1.199559e-03	0.1580
6	0.1678	0.757000	0.756894	1.063536e-04	0.0140
7	0.2132	0.757000	0.755986	1.014321e-03	0.1340
8	0.2545	0.755500	0.754970	5.304707e-04	0.0702
9	0.2924	0.754000	0.753583	4.168800e-04	0.0553
10	0.3269	0.750500	0.751340	−8.403851e-04	0.1120
11	0.3585	0.746500	0.747329	−8.294717e-04	0.1111
12	0.3873	0.738500	0.740096	−1.595761e-03	0.2161
13	0.4137	0.728000	0.727391	6.087357e-04	0.0836
14	0.4373	0.706500	0.706907	−4.070601e-04	0.0576
15	0.4590	0.675500	0.675158	3.420485e-04	0.0506
16	0.4784	0.632000	0.630608	1.392162e-03	0.2203
17	0.4960	0.573000	0.571633	1.367006e-03	0.2386
18	0.5119	0.499000	0.498870	1.297571e-04	0.0260
19	0.5265	0.413000	0.412736	2.644958e-04	0.0640
20	0.5398	0.316500	0.316394	1.062714e-04	0.0336
21	0.5521	0.212000	0.211297	7.026340e-04	0.3314
22	0.5633	0.103500	0.102028	1.471969e-03	1.4222
23	0.5736	−0.010000	−0.009743	−2.570082e-04	2.5701
24	0.5833	−0.123000	−0.124594	1.594214e-03	1.2961
25	0.5900	−0.210000	−0.209155	−8.446127e-04	0.4022

**Table 8:** RIO-ML algorithm – RMSE statistics for 30 independent runs (SDM).

Run	Max RMSE	Min RMSE	Mean RMSE	Std RMSE	Variance RMSE
1	8.248871e-02	8.862001e-04	1.102722e-02	1.159861e-02	1.345278e-04
2	1.177319e-01	1.436853e-03	9.643353e-03	1.467911e-02	2.154763e-04
3	1.514931e-01	1.657271e-03	1.231967e-02	1.771577e-02	3.138485e-04
4	1.687534e-01	9.339071e-04	1.253268e-02	2.286442e-02	5.227817e-04
5	5.763992e-02	2.140676e-03	1.155227e-02	1.195774e-02	1.429876e-04
6	1.771526e-01	1.156149e-03	1.413431e-02	2.053173e-02	4.215521e-04
7	1.093934e-01	2.444602e-03	1.448770e-02	1.684020e-02	2.835923e-04
8	1.543972e-01	2.519084e-03	1.120672e-02	1.470938e-02	2.163659e-04
9	1.600849e-01	1.346149e-03	1.479234e-02	2.014839e-02	4.059576e-04
10	1.927181e-01	2.695286e-03	6.614865e-03	1.305230e-02	1.703626e-04
11	1.119460e-01	7.984237e-03	1.237401e-02	8.787056e-03	7.721235e-05
12	1.725904e-01	1.815243e-03	8.101702e-03	1.406005e-02	1.976849e-04
13	1.566687e-01	1.544416e-03	1.291765e-02	1.667452e-02	2.780395e-04
14	1.554099e-01	1.651123e-03	1.330160e-02	1.867025e-02	3.485784e-04
15	1.680889e-01	1.938247e-03	1.241676e-02	2.141302e-02	4.585176e-04
16	1.470955e-01	8.917552e-04	8.257292e-03	1.780217e-02	3.169173e-04
17	1.381460e-01	1.200384e-03	1.484802e-02	2.071938e-02	4.292929e-04
18	2.047514e-01	2.982544e-03	1.186942e-02	1.685260e-02	2.840102e-04
19	8.412707e-02	2.271728e-03	1.417990e-02	1.607107e-02	2.582794e-04
20	1.678459e-01	1.577944e-03	9.722334e-03	1.709685e-02	2.923022e-04
21	6.844546e-02	3.827343e-03	9.949579e-03	9.371120e-03	8.781788e-05
22	8.723828e-02	2.088396e-03	9.686987e-03	1.535622e-02	2.358136e-04
23	1.496175e-01	1.092000e-03	8.863854e-03	1.546273e-02	2.390961e-04
24	7.339632e-02	4.220474e-03	1.184317e-02	9.997905e-03	9.995809e-05
25	1.750350e-01	1.943012e-03	1.115303e-02	1.878794e-02	3.529868e-04
26	1.702489e-01	4.356905e-03	1.444757e-02	1.958751e-02	3.836704e-04
27	1.622997e-01	3.295073e-03	1.012484e-02	1.739219e-02	3.024884e-04
28	1.418784e-01	2.441440e-03	8.369705e-03	1.417677e-02	2.009809e-04
29	1.943769e-01	1.480850e-03	1.349308e-02	2.149498e-02	4.620342e-04
30	1.044932e-01	8.739710e-04	9.984917e-03	1.266156e-02	1.603152e-04

**Fig. 6:**
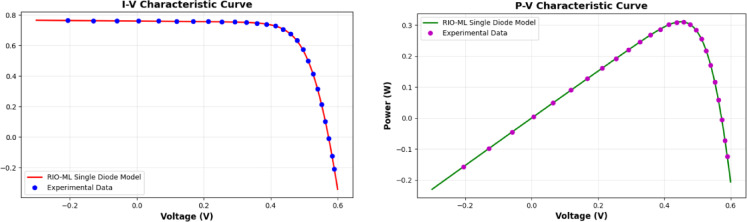
Measured and estimated I–V and P-V curves of RIO-ML algorithm for SDM.

**Fig. 7:**
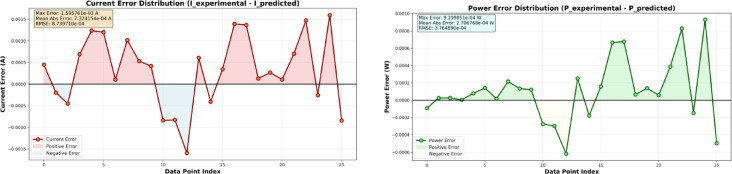
Current and power error distribution of RIO-ML algorithm for SDM.

**Fig. 8:**
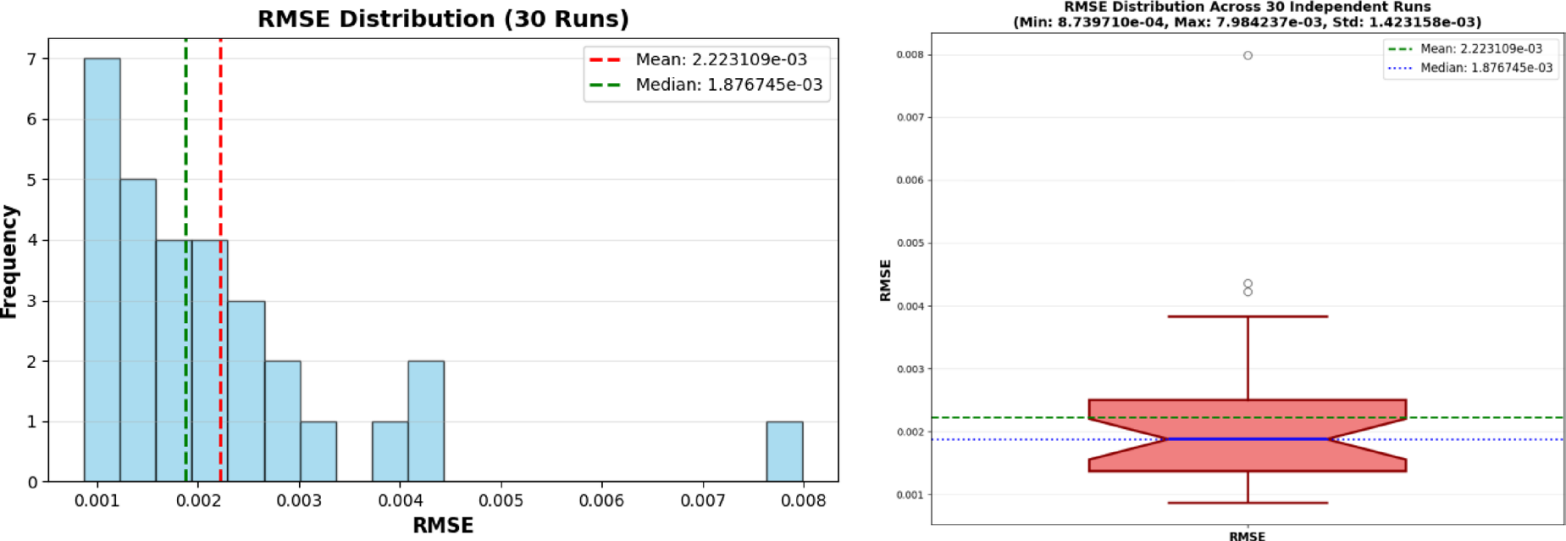
RMSE distribution and box plot of RIO-ML for SDM over 30 independent runs.

Figure 6 illustrate the measured and estimated I-V and P-V characteristic curves, respectively, showing excellent visual agreement between experimental data and model predictions across the entire operating range. Figures 7 and 8 show the, current error distribution, power error distribution, RMSE distribution and Box plot of RMSE across over 30 runs, confirming the robustness and consistency of the optimization process.

**Fig. 9:**
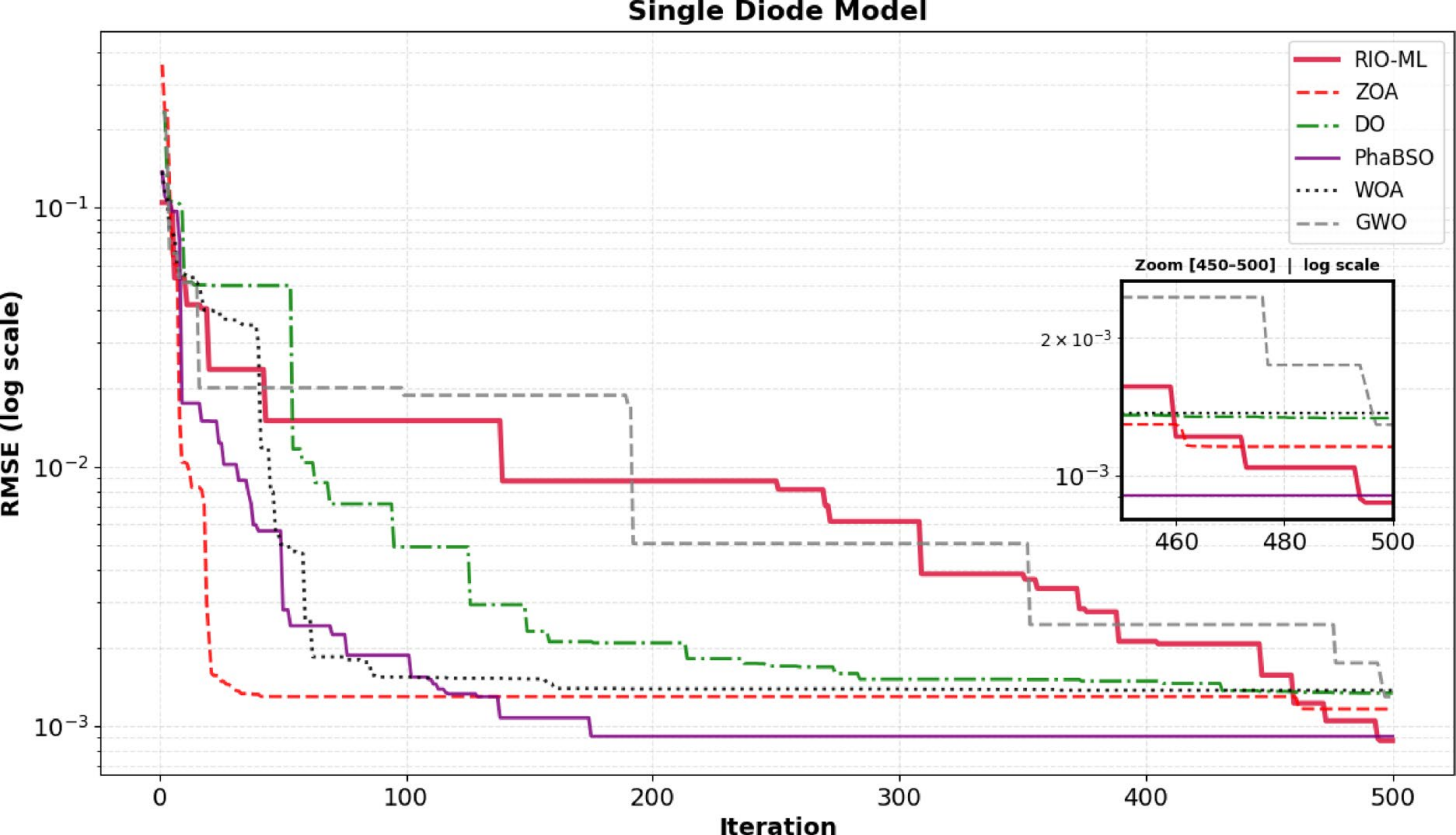
The convergence curve of RIO-ML algorithm versus other algorithms for SDM.

The comparative analysis of the convergence curves for the RIO-ML algorithm is evaluated against ZOA, DO, PhaBSO, WOA, and GWO for SDM, as shown in Fig. 9. The results demonstrate that RIO-ML maintains a superior balance between exploration and exploitation. RIO-ML shows constant progress in reducing RMSE until it achieves its lowest final error, while ZOA, WOA, and PhaBSO display quick initial descent, which leads to early performance stagnation. RIO-ML shows superior performance to competitors during the last fine-tuning step, and the Zoom [450–500] inset shows in magnified detail. Also the adaptive learning mechanism achieved its highest precision in parameter extraction through RIO-ML, which shows superior performance to competitors during the last fine-tuning step.

### Double diode model results

The Double Diode Model (DDM) with 7 parameters is challenging to optimize due to higher dimensions and interactions between parameters. Despite this complexity, RIO-ML achieved outstanding results with a minimum RMSE of 8.456760e-04 in Run 17, which is even lower than the best SDM result, as shown in Table 12. Table 9 presents the statistical summary of the RMSE results statistical analysis for RIO-ML and other algorithms. the results demonstrate the lower RMSE of the proposed RIO-ML approach. In addition, A mean RMSE of the proposed RIO-ML is 2.282687e-03 with a standard deviation of 1.444465e-03 across 30 independent runs. And the low variance (2.086480e-06) of the RIO-ML confirms the algorithm’s stability and reliability even with the increased model complexity.

The optimal parameters of DDM are identified by the best run for RIO-ML, and other algorithms are presented in the Table 10.

**Table 9:** Statistical metrics of RIO-ML, ZOA, DO, PhaBSO, WOA, and GWO for DDM.

RMSE	Optimization algorithms					
	RIO-ML	ZOA	DO	PhaBSO	WOA	GWO
Min	8.456760e-04	1.1111976e-03	1.1397497e-03	8.935648e-04	1.208910e-03	1.2713636e-03
Mean	2.282687e-03	2.3523995e-03	2.8012064e-03	2.570058e-03	1.050234e-02	9.3054570e-03
Max	5.969559e-03	7.5583407e-03	9.0292519e-03	6.262033e-03	5.105890e-02	4.3191362e-02
Median	1.699351e-03	2.1762749e-03	2.2832856e-03	2.451019e-03	5.691416e-03	3.7935262e-03
Std	1.444465e-03	1.2580062e-03	1.6944445e-03	1.112882e-03	1.195755e-02	1.3413209e-02
Variance	2.086480e-06	1.5825795e-06	2.8711421e-06	1.238507e-06	1.429830e-04	1.7991418e-04

**Table 10:** Optimal parameters of RIO-ML, ZOA, DO, PhaBSO, WOA, and GWO for DDM.

Parameter	LB	UB	Optimal parameters of optimization algorithms					
			RIO-ML	ZOA	DO	PhaBSO	WOA	GWO
Iph (A)	0	1	0.760373	0.76146907	0.76002358	0.760889	0.760106	0.76189000
Isd1 (A)	0	1e-6	7.460648e-07	1.5134e-07	3.7741e-07	2.654418e-07	3.403572e-07	7.7316e-07
Isd2 (A)	0	1e-6	1.495416e-07	3.5926e-07	1.8349e-09	1.876408e-07	8.625106e-08	1.9551e-07
Rs (Ω)	0	0.5	0.036888	0.03648942	0.03587522	0.035374	0.035913	0.03675827
Rsh (Ω)	0	100	64.675290	46.57370596	71.62690523	58.654264	79.656542	47.45558135
n1	1	2	1.811189	1.42966219	1.49691048	1.481470	1.500690	1.90346939
n2	1	2	1.423156	1.67499121	1.73139376	1.609066	1.556024	1.44138202

Table 11 compares experimental and predicted current values for all 26 measurement points. The DDM achieves a maximum absolute error of 1.742920e-03 A and mean absolute error of 6.938742–04 A, representing improved accuracy over the SDM (Table 12).

**Table 11:** Experimental vs. predicted current of double diode model (Best run).

Index	Voltage (V)	I_exp (A)	I_pred (A)	Error (A)	Error %
0	−0.2057	0.764000	0.763119	8.807427e-04	0.1153
1	−0.1291	0.762000	0.761935	6.452647e-05	0.0085
2	−0.0588	0.760500	0.760849	−3.487671e-04	0.0459
3	0.0057	0.760500	0.759851	6.493737e-04	0.0854
4	0.0646	0.760000	0.758935	1.064618e-03	0.1401
5	0.1185	0.759000	0.758086	9.139757e-04	0.1204
6	0.1678	0.757000	0.757275	−2.752852e-04	0.0364
7	0.2132	0.757000	0.756438	5.617670e-04	0.0742
8	0.2545	0.755500	0.755457	4.282220e-05	0.0057
9	0.2924	0.754000	0.754060	−5.953341e-05	0.0079
10	0.3269	0.750500	0.751752	−1.251934e-03	0.1668
11	0.3585	0.746500	0.747625	−1.124830e-03	0.1507
12	0.3873	0.738500	0.740243	−1.742920e-03	0.2360
13	0.4137	0.728000	0.727394	6.060074e-04	0.0832
14	0.4373	0.706500	0.706818	−3.177293e-04	0.0450
15	0.4590	0.675500	0.675062	4.376295e-04	0.0648
16	0.4784	0.632000	0.630600	1.400089e-03	0.2215
17	0.4960	0.573000	0.571782	1.218477e-03	0.2126
18	0.5119	0.499000	0.499200	−1.997598e-04	0.0400
19	0.5265	0.413000	0.413232	−2.317244e-04	0.0561
20	0.5398	0.316500	0.317015	−5.145190e-04	0.1626
21	0.5521	0.212000	0.211993	6.763181e-06	0.0032
22	0.5633	0.103500	0.102752	7.477053e-04	0.7224
23	0.5736	−0.010000	−0.009026	−9.740946e-04	9.7409
24	0.5833	−0.123000	−0.123909	9.092882e-04	0.7393
25	0.5900	−0.210000	−0.208504	−1.495846e-03	0.7123

**Table 12:** RMSE statistics for 30 independent runs (Double-diode model).

Run	Max RMSE	Min RMSE	Mean RMSE	Std RMSE	Variance RMSE
1	1.320735e-01	5.969559e-03	1.363593e-02	1.577294e-02	2.487856e-04
2	1.174833e-01	2.529125e-03	7.905608e-03	8.336587e-03	6.949868e-05
3	1.548584e-01	2.010145e-03	1.363359e-02	1.472927e-02	2.169513e-04
4	1.392569e-01	2.657311e-03	1.242217e-02	1.916857e-02	3.674342e-04
5	1.357542e-01	3.489180e-03	1.199145e-02	1.171573e-02	1.372583e-04
6	1.012424e-01	1.565511e-03	9.194786e-03	1.135058e-02	1.288356e-04
7	1.121603e-01	2.856464e-03	1.099938e-02	1.435995e-02	2.062082e-04
8	1.455384e-01	1.244568e-03	1.069418e-02	1.419750e-02	2.015689e-04
9	8.730067e-02	1.627999e-03	1.014883e-02	1.168342e-02	1.365023e-04
10	1.710478e-01	1.172202e-03	1.178486e-02	1.689820e-02	2.855491e-04
11	1.555183e-01	1.127678e-03	1.096347e-02	1.647838e-02	2.715371e-04
12	1.092026e-01	2.726755e-03	1.004517e-02	8.998908e-03	8.098035e-05
13	1.225941e-01	1.004346e-03	6.307427e-03	8.338842e-03	6.953628e-05
14	1.258400e-01	2.239665e-03	1.706590e-02	1.594020e-02	2.540899e-04
15	2.101301e-01	2.650474e-03	1.025062e-02	1.385453e-02	1.919479e-04
16	1.622620e-01	1.770702e-03	1.475982e-02	1.529038e-02	2.337958e-04
17	1.163785e-01	8.456760e-04	1.047755e-02	1.391855e-02	1.937261e-04
18	1.444493e-01	1.935308e-03	1.365292e-02	1.469669e-02	2.159928e-04
19	2.043622e-01	3.156449e-03	9.876391e-03	1.331238e-02	1.772193e-04
20	1.495009e-01	1.480520e-03	1.203235e-02	2.014265e-02	4.057263e-04
21	1.176421e-01	4.073160e-03	1.229978e-02	1.384930e-02	1.918032e-04
22	1.888658e-01	1.217614e-03	7.143281e-03	1.214419e-02	1.474814e-04
23	2.090213e-01	5.966439e-03	1.557645e-02	1.558444e-02	2.428748e-04
24	1.598324e-01	9.211863e-04	1.090102e-02	1.388059e-02	1.926709e-04
25	4.643717e-02	1.318430e-03	9.078016e-03	8.430499e-03	7.107332e-05
26	1.492247e-01	1.239558e-03	1.083762e-02	1.736266e-02	3.014621e-04
27	1.945059e-01	5.727936e-03	1.699768e-02	1.944047e-02	3.779317e-04
28	1.503863e-01	1.338085e-03	1.378394e-02	1.856856e-02	3.447914e-04
29	4.772093e-02	1.522916e-03	1.002605e-02	1.228048e-02	1.508103e-04
30	1.157191e-01	1.095655e-03	8.073527e-03	1.375783e-02	1.892778e-04

**Fig. 10:**
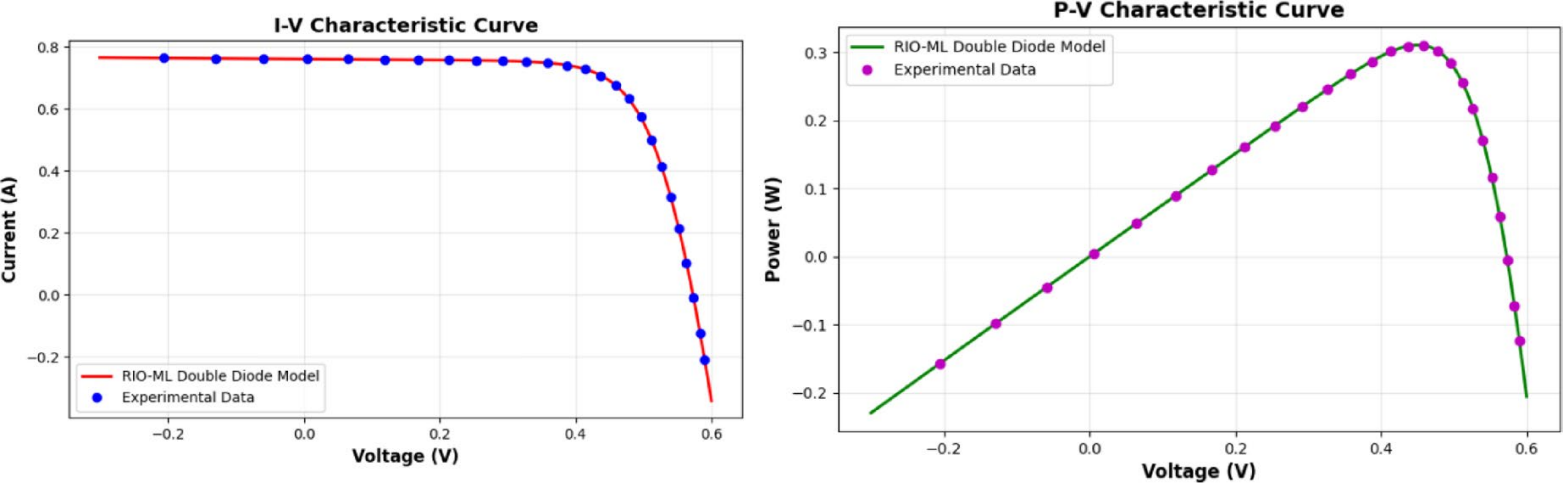
Measured and estimated I–V and P-V curves of RIO-ML algorithm for DDM.

**Fig. 11:**
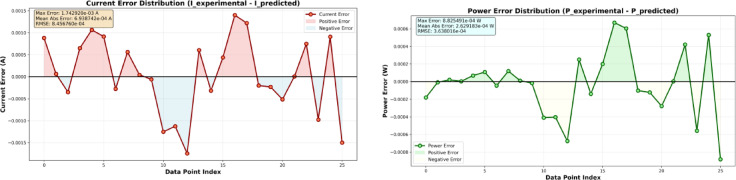
Current and power error distribution of RIO-ML algorithm for DDM.

**Fig. 12:**
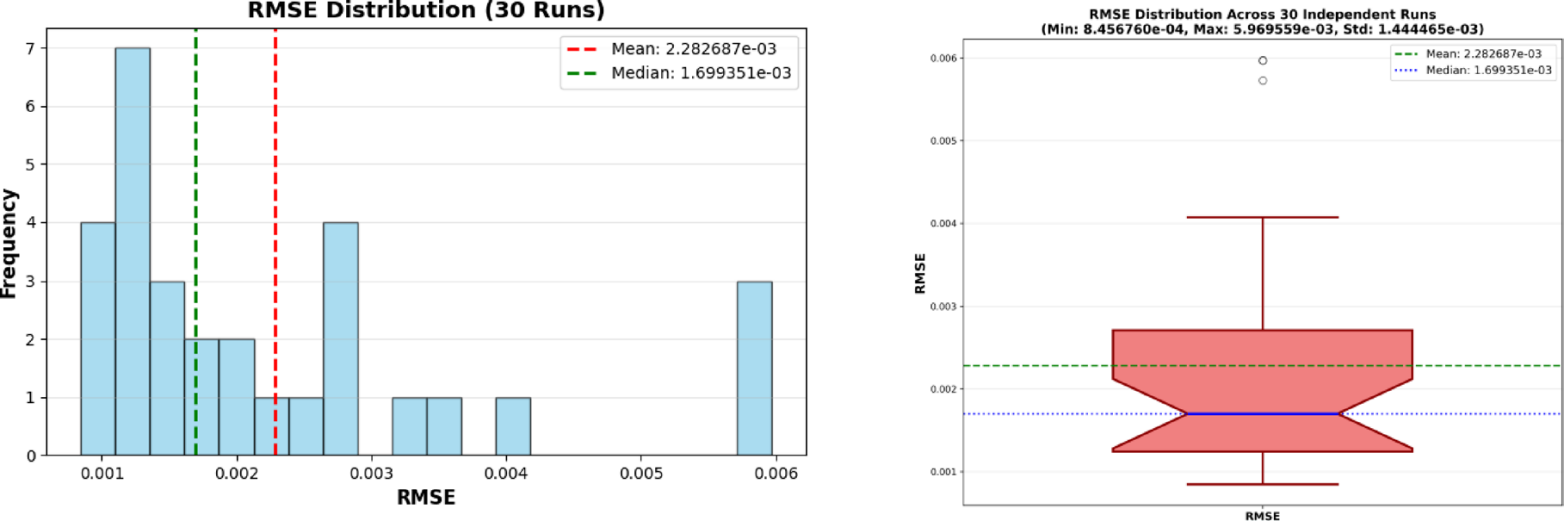
RMSE distribution and box plot of RIO-ML for DDM over 30 independent runs.

The Visual results for I-V, and P-V curves characteristic curves, respectively, showing excellent visual agreement between experimental data and model predictions across the entire operating range, as shown in Fig. 10. Figures 11 and 12 show the, current error distribution, power error distribution, RMSE distribution and Box plot of RMSE across over 30 runs, demonstrating the algorithm’s capability to handle high-dimensional optimization problems effectively.

**Fig. 13:**
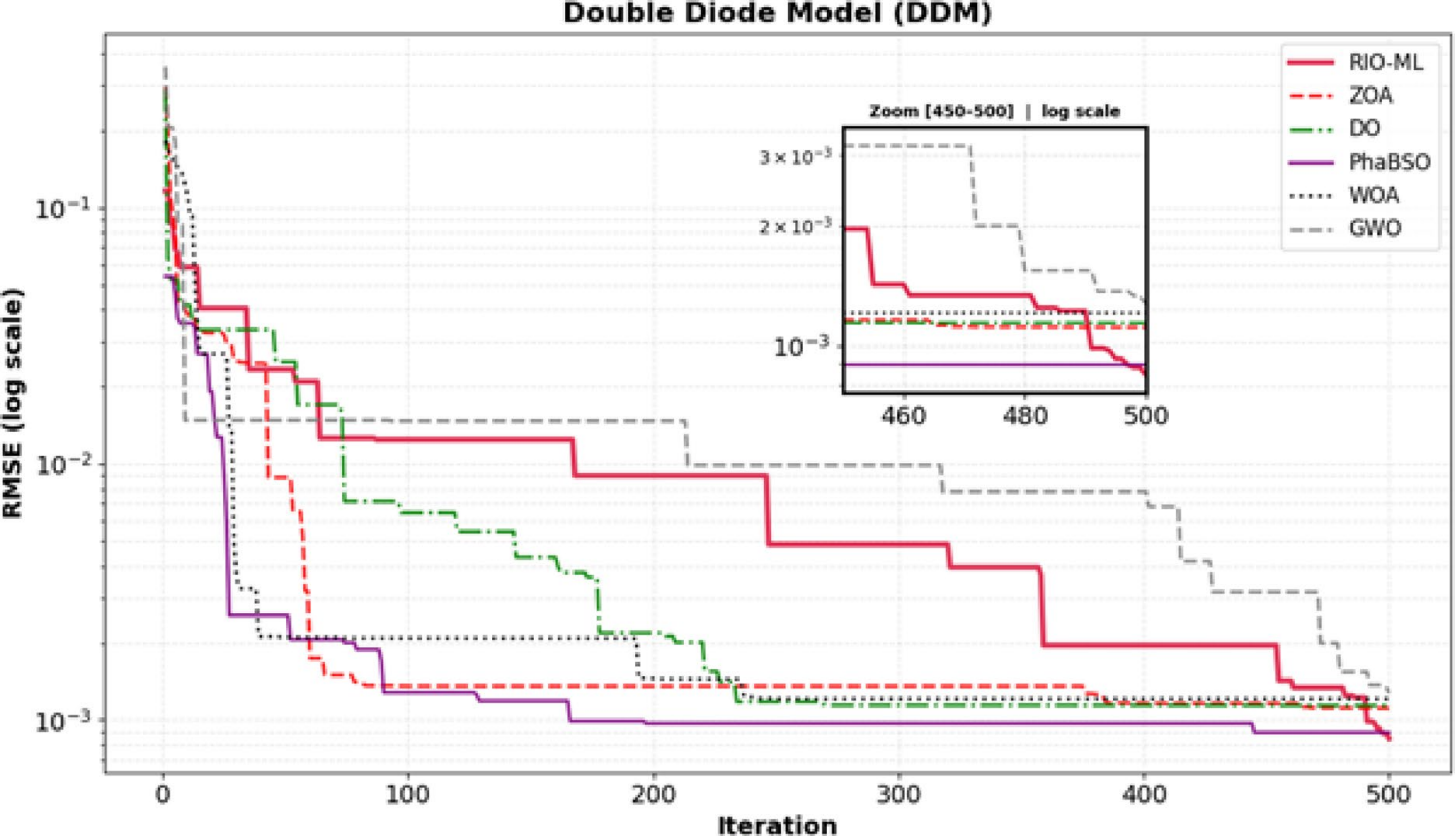
The convergence curve of RIO-ML algorithm versus other algorithms for DDM.

The comparative analysis of the convergence curves for the proposed RIO-ML algorithm is compared with ZOA, DO, PhaBSO, WOA, and GWO for the DDM, as explained in Fig. 13. The obtained results indicate that RIO-ML achieves an effective balance between exploration and exploitation throughout the optimization process. Unlike ZOA, WOA, DO, and PhaBSO, which exhibit rapid initial convergence followed by early stagnation, RIO-ML continues to gradually reduce the RMSE until reaching the lowest final error value. the Zoom [450–500] highlights the superior fine-tuning capability of RIO-ML during the final optimization stage. Also the adaptive learning mechanism achieved its highest precision in parameter extraction through RIO-ML, which shows superior performance to competitors during the last fine-tuning step.

### Triple diode model results

The TDM poses nine parameters, represents the complicated optimization problem in this study. It is considered the final challenging benchmark to assess the full capability of the RIO-ML algorithm. The proposed RIO-ML model demonstrated outstanding performance in Run 22, with a minimum RMSE of 7.754698e-04 A, despite the complexity involved, as shown in Table 16. the statistical summary of the RMSE results for RIO-ML and other algorithms, which indicates the performance of the proposed RIO-ML even in high-dimensional search spaces, is presented in Table 13 with a mean RMSE of 1.717649e-03, a standard deviation of 9.398510e-04, and the low variance (8.833200e-07) confirms the algorithm’s stability and reliability even with the increased model complexity.

The optimal parameters of the TDM obtained from the best run of RIO-ML and the competing algorithms are presented in Table 14.

**Table 13:** Statistical metrics of RIO-ML, ZOA, DO, PhaBSO, WOA, and GWO for TDM.

Statistic	Optimization algorithms					
	RIO-ML	ZOA	DO	PhaBSO	WOA	GWO
Min	7.754698e-04	1.0175176e-03	1.0353307e-03	8.405556e-04	9.579256e-04	1.2357999e-03
Mean	1.717649e-03	2.9492416e-03	3.0921694e-03	2.414941e-03	9.833907e-03	7.4220851e-03
Max	4.744082e-03	9.2454477e-03	5.6707749e-03	3.781699e-03	3.784992e-02	4.0323896e-02
Median	1.323858e-03	2.5148728e-03	3.0820674e-03	2.253801e-03	4.935904e-03	2.4672844e-03
Std	9.398510e-04	1.6682595e-03	1.0101053e-03	7.113981e-04	8.662023e-03	1.1110570e-02
Variance	8.833200e-07	2.78308966e-06	1.0211816e-06	5.060872e-07	7.503064e-05	1.2344476e-04

**Table 14:** Optimal Parameters of RIO-ML, ZOA, DO, PhaBSO, WOA, and GWO for TDM.

Parameter	LB	UB	Optimal parameters of optimization algorithms					
			RIO-ML	ZOA	DO	PhaBSO	WOA	GWO
Iph (A)	0	1	0.760814	0.76047006	0.76118277	0.760433	0.760654	0.76185637
Isd1 (A)	0	1e-6	2.448328e-07	2.7811e-07	2.0125e-07	1.782263e-07	4.710310e-07	5.4147e-08
Isd2 (A)	0	1e-6	1.031167e-07	1.6728e-07	9.1624e-08	1.836730e-07	1.301806e-10	2.9302e-07
Isd3 (A)	0	1e-6	2.176607e-07	5.2474e-08	2.1606e-07	3.265585e-07	2.092467e-07	6.7281e-09
Rs (Ω)	0	0.5	0.037533	0.03685067	0.03687484	0.036501	0.035434	0.03637380
Rsh (Ω)	0	100	52.588982	60.70748871	47.74600407	61.610619	56.178169	41.95330923
n1	1	2	1.793689	1.63954294	1.52011353	1.638221	1.737782	1.96771954
n2	1	2	1.398598	1.54217621	1.41648075	1.442520	1.401751	1.47304643
n3	1	2	1.625725	1.37525160	1.97772226	1.984565	1.456414	1.57017431

Table 15 shows experimental versus predicted currents for the best run for all 26 measurement points. The model achieves a maximum absolute error of 1.448958e-03 A and mean absolute error of 6.74255e-04, maintaining excellent accuracy over the SDM and DDM, despite the model complexity (Table 16).

**Table 15:** Experimental vs. predicted current of TDM for RIO-ML algorithm.

Index	Voltage (V)	I_exp (A)	I_pred (A)	Error (A)	Error %
0	−0.2057	0.764000	0.764181	−1.809986e-04	0.0237
1	−0.1291	0.762000	0.762725	−7.254118e-04	0.0952
2	−0.0588	0.760500	0.761389	−8.893581e-04	0.1169
3	0.0057	0.760500	0.760163	3.372258e-04	0.0443
4	0.0646	0.760000	0.759040	9.601250e-04	0.1263
5	0.1185	0.759000	0.758003	9.970636e-04	0.1314
6	0.1678	0.757000	0.757026	−2.624838e-05	0.0035
7	0.2132	0.757000	0.756048	9.518004e-04	0.1257
8	0.2545	0.755500	0.754961	5.389028e-04	0.0713
9	0.2924	0.754000	0.753504	4.957237e-04	0.0657
10	0.3269	0.750500	0.751201	−7.013706e-04	0.0935
11	0.3585	0.746500	0.747159	−6.589744e-04	0.0883
12	0.3873	0.738500	0.739949	−1.448958e-03	0.1962
13	0.4137	0.728000	0.727347	6.528274e-04	0.0897
14	0.4373	0.706500	0.707042	−5.422498e-04	0.0768
15	0.4590	0.675500	0.675518	−1.840388e-05	0.0027
16	0.4784	0.632000	0.631168	8.318158e-04	0.1316
17	0.4960	0.573000	0.572306	6.939240e-04	0.1211
18	0.5119	0.499000	0.499539	−5.387228e-04	0.1080
19	0.5265	0.413000	0.413298	−2.980443e-04	0.0722
20	0.5398	0.316500	0.316799	−2.991328e-04	0.0945
21	0.5521	0.212000	0.211552	4.482064e-04	0.2114
22	0.5633	0.103500	0.102190	1.310305e-03	1.2660
23	0.5736	−0.010000	−0.009586	−4.137079e-04	4.1371
24	0.5833	−0.123000	−0.124338	1.338100e-03	1.0879
25	0.5900	−0.210000	−0.208757	−1.243035e-03	0.5919

**Table 16:** RMSE statistics for 30 independent runs of TDM for RIO-ML.

Run	Max RMSE	Min RMSE	Mean RMSE	Std RMSE	Variance RMSE
1	1.247530e-01	2.043826e-03	9.104517e-03	1.110132e-02	1.232393e-04
2	1.288150e-01	8.001841e-04	6.063059e-03	1.027178e-02	1.055095e-04
3	1.291875e-01	4.744082e-03	9.018387e-03	8.561293e-03	7.329574e-05
4	4.358468e-02	1.362421e-03	7.282243e-03	7.732004e-03	5.978388e-05
5	1.542472e-01	2.125270e-03	7.081604e-03	1.110023e-02	1.232151e-04
6	7.066716e-02	3.150681e-03	5.826735e-03	9.309811e-03	8.667259e-05
7	1.013762e-01	2.788643e-03	6.486028e-03	8.179880e-03	6.691043e-05
8	1.659743e-01	2.342614e-03	8.684314e-03	1.264344e-02	1.598565e-04
9	2.130212e-01	1.177572e-03	5.727671e-03	1.083880e-02	1.174797e-04
10	9.500189e-02	2.027575e-03	7.785376e-03	1.056508e-02	1.116209e-04
11	1.132519e-01	9.885237e-04	4.167758e-03	7.330220e-03	5.373213e-05
12	1.089564e-01	1.067953e-03	7.347106e-03	1.118537e-02	1.251126e-04
13	8.063972e-02	2.090438e-03	7.306835e-03	7.296660e-03	5.324125e-05
14	1.383639e-01	3.870296e-03	9.654938e-03	1.266601e-02	1.604279e-04
15	1.108937e-01	2.491080e-03	8.341197e-03	1.005737e-02	1.011506e-04
16	1.494175e-01	8.349106e-04	8.275341e-03	1.180212e-02	1.392900e-04
17	1.108986e-01	1.166200e-03	5.999810e-03	7.661632e-03	5.870060e-05
18	1.441337e-01	8.779401e-04	7.466250e-03	1.109442e-02	1.230863e-04
19	1.428991e-01	1.995956e-03	9.205981e-03	1.332651e-02	1.775959e-04
20	1.907017e-01	1.282969e-03	1.020874e-02	1.356520e-02	1.840147e-04
21	1.212141e-01	9.221226e-04	3.985782e-03	7.388552e-03	5.459070e-05
22	1.765457e-01	7.754698e-04	6.974245e-03	1.140617e-02	1.301008e-04
23	8.393262e-02	8.595732e-04	5.893376e-03	7.555499e-03	5.708557e-05
24	1.151332e-01	1.285294e-03	6.114180e-03	7.209283e-03	5.197377e-05
25	8.742236e-02	1.928112e-03	7.865691e-03	7.823839e-03	6.121246e-05
26	8.946828e-02	1.125090e-03	6.376288e-03	7.834703e-03	6.138257e-05
27	1.819744e-01	1.475827e-03	7.995157e-03	1.161218e-02	1.348427e-04
28	1.154352e-01	9.442358e-04	6.259309e-03	8.720401e-03	7.604539e-05
29	9.958376e-02	1.224104e-03	5.472645e-03	9.631096e-03	9.275800e-05
30	1.032125e-01	1.760504e-03	7.502076e-03	1.066249e-02	1.136886e-04

**Fig. 14:**
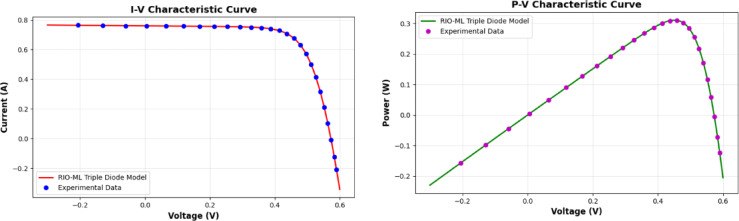
Measured and estimated I–V and P-V curves of RIO-ML algorithm for TDM.

**Fig. 15:**
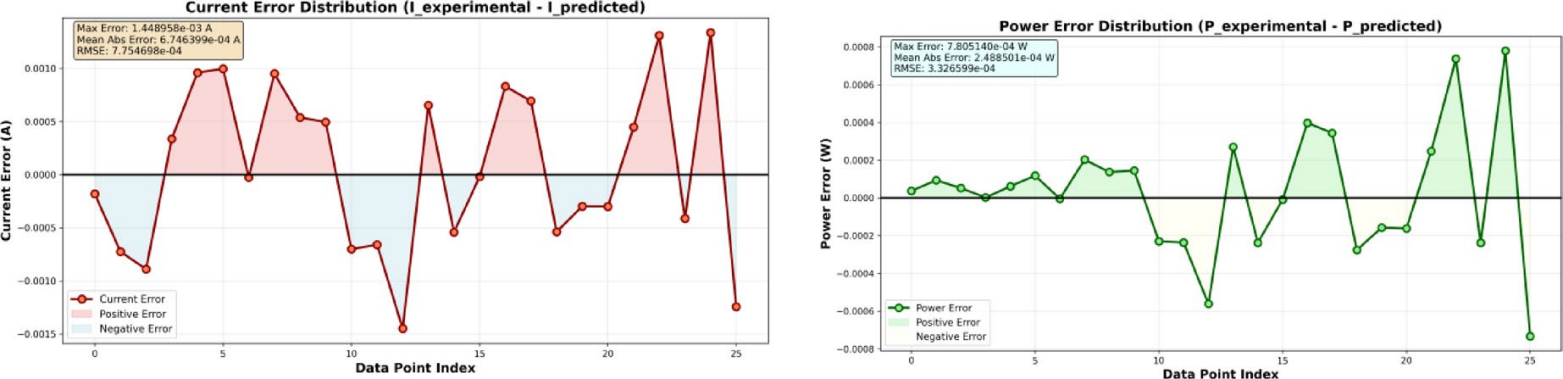
Current and power error distribution of RIO-ML algorithm for TDM.

**Fig. 16:**
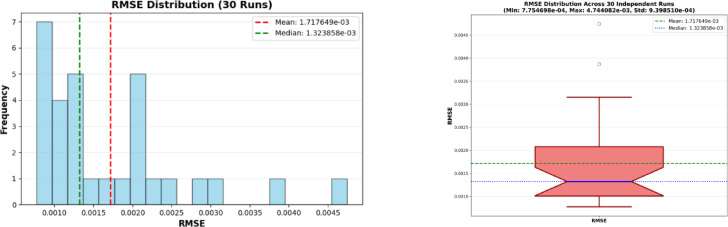
RMSE distribution and box plot of RIO-ML for TDM over 30 independent runs.

The Visual results for I-V, and P-V curves characteristic curves, respectively, showing excellent visual agreement between experimental data and model predictions across the entire operating range, as shown in Fig. 14. Figures 15 and 16 show the, current error distribution, power error distribution, RMSE distribution and Box plot of RMSE across over 30 runs, demonstrating the algorithm’s capability to handle high-dimensional optimization problems effectively.

**Fig. 17:**
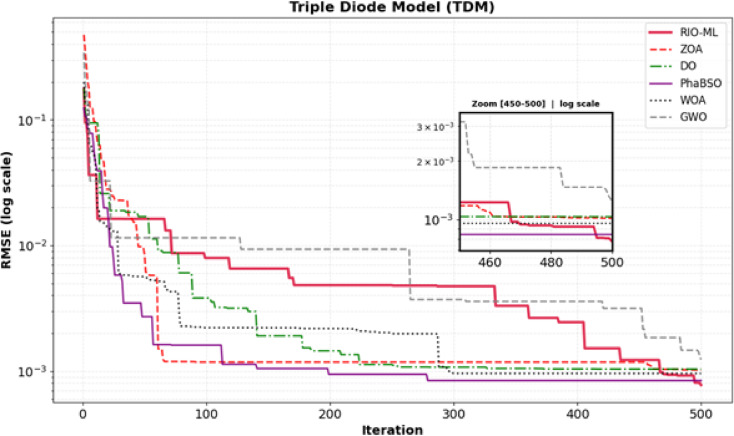
The convergence curve of RIO-ML algorithm versus other ealgorithms for TDM.

The comparative analysis of the convergence curves for the proposed RIO-ML algorithm is compared with ZOA, DO, PhaBSO, WOA, and GWO for the DDM, as explained in Fig. 17. The obtained results indicate that RIO-ML achieves an effective balance between exploration and exploitation throughout the optimization process. Unlike ZOA, WOA, DO, and PhaBSO, which exhibit rapid initial convergence followed by early stagnation, RIO-ML continues to gradually reduce the RMSE until reaching the lowest final error value. the Zoom [450–500] highlights the superior fine-tuning capability of RIO-ML during the final optimization stage. Also the adaptive learning mechanism achieved its highest precision in parameter extractionthrough RIO-ML, which shows superior performance to competitors during the last fine-tuning step.

### Comparative analysis and statistical validation

RIO-ML demonstrates superior performance compared to recent state-of-the-art algorithms on the RTC France dataset. For SDM, our minimum RMSE of **8.739710e-04** outperforming ZOA (1.1582255e-03), DO (1.3352770e-03), PhaBSO (9.084225e-04), WOA (1.368912e-03), and GWO (1.2917508e-03); similar for DDM, RIO-ML again yields the best performance with an RMSE of **8.456760e-04**, compared to ZOA (1.1111976e-03), DO (1.1397497e-03), PhaBSO (8.935648e-04), WOA (1.208910e-03), and GWO (1.2713636e-03). TDM, RIO-ML maintains its superiority with a minimum RMSE of **7.754698e-04**, while ZOA, DO, PhaBSO, WOA, and GWO achieve 1.0175176e-03, 1.0353307e-03, 8.405556e-04, 9.579256e-04, and 1.2357999e-03, respectively.

The computational complexity of RIO-ML is,$\:O\left(\left({T}_{max}\times\:N\times\:\mathrm{dim}\right)+\left({ML}_{train}\right)\right)$, where T is the maximum number of iterations, N is the population size, D is the search space dimensionality, H is the number of hidden neurons (25), and F is the feature dimension (3). The first term $\:O\left(\left({T}_{max}\times\:N\times\:\mathrm{dim}\right)\right)$ is identical to all compared methods, while the second term ($\:O\left({ML}_{train}\right)=O\left(H\times\:F\right)$) represents a constant, bounded overhead introduced by the MLP training step, which does not scale with N or D.

**Fig. 18:**
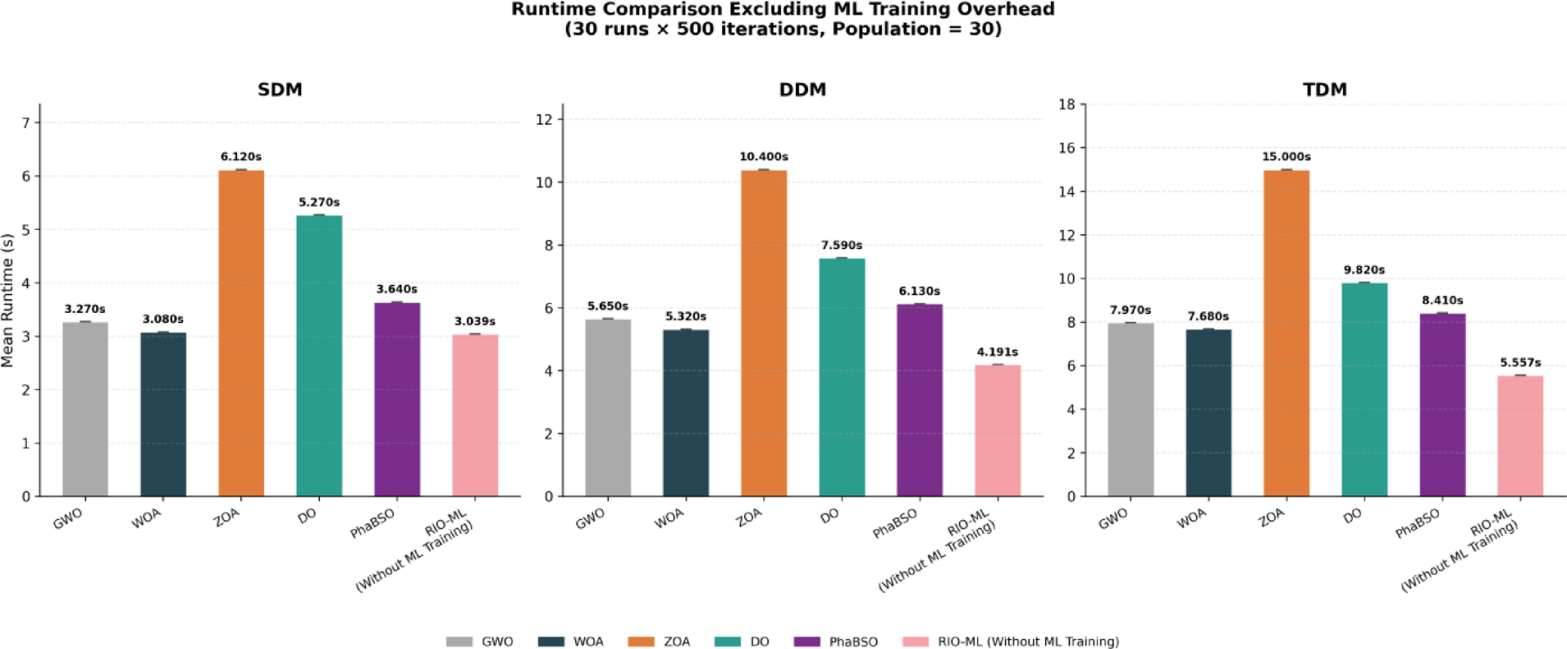
Average computational time per run for the compared optimization algorithms across three PV models (30 independent runs, 500 iterations, population size = 30).

The mean computational runtime was measured for all algorithms(RIO-ML, ZOA, DO, PhaBSO, WOA, and GWO) across the SDM, DDM, and TDM models under conditions, as detailed in Table 3, with results reported in Table 17 and illustrated in Fig. 18. The total mean runtime of RIO-ML (including ML training) is 46.67 s, 48.84 s, and 50.32 s for SDM, DDM, and TDM, respectively, of which the ML training component accounts for 43.63 s, 44.65 s, and 44.76 s. When the ML training overhead is excluded, the core optimization runtime of RIO-ML reduces to 3.039 s, 4.191 s, and 5.557 s for SDM, DDM, and TDM, respectively, which is not only comparable to but lower than most competing methods, including ZOA (6.12 s, 10.40 s, 15.00 s) and DO (5.27 s, 7.59 s, 9.82 s), and competitive with GWO, WOA, and PhaBSO across all three models.

The ML training overhead is a one-time bounded cost per run that does not scale with N or D, and is explicitly justified by the superior solution accuracy and convergence quality delivered by RIO-ML. The adaptive MLP mechanism enables RIO-ML to dynamically balance exploration and exploitation throughout the search process a capability unavailable in fixed-parameter methods resulting in strictly lower final RMSE values across all three models and all 30 independent runs, as confirmed by the statistical results reported in Tables 5 and 9, and 13, and further supported by the convergence curves illustrated in Figs. 9 and 13, and 17.

**Table 17:** Mean runtime of the optimization algorithms for the SDM, DDM, and TDM models obtained from 30 independent runs with 500 iterations per run.

Algorithm	Mean time (s)		
	SDM	DDM	TDM
GWO	3.27	5.65	7.97
WOA	3.08	5.32	7.68
ZOA	6.12	10.40	15.00
DO	5.27	7.59	9.82
PhaBSO	3.64	6.13	8.41
RIO-ML (without Ml training)	3.039	4.191	5.557
ML training	43.631	44.649	44.763
RIO-ML	46.67	48.84	50.32

## Conclusion

The proposed work introduces a new hybrid meta-heuristic technique for the estimation of photovoltaic parameters, which is called the Reflective Intelligence Optimizer with Machine Learning (RIO-ML). The proposed technique consists of three new components: progressive Gaussian refinement with reflective boundary handling, machine learning-based adaptive parameter adjustment using MLP neural networks, and multi-leader social learning with individual best memory. The proposed technique is thoroughly validated on the RTC France solar cell dataset and proves its effectiveness through 30 independent runs for three different model configurations. RIO-ML demonstrates superior performance compared to another algorithms on the RTC France dataset. For SDM, our minimum RMSE of 8.739710e-04 A; similar for DDM, RIO-ML again yields the best performance with an RMSE of 8.456760e-04 A. In TDM, RIO-ML maintains its superiority with a minimum RMSE of 7.754698e-04 A. The absolute current errors at their maximum do not exceed 1.6e-03 A for all models, indicating an excellent I-V curve reconstruction across the entire range of operation The MLP neural networks adaptively learn the optimal values of the social-reflective balance coefficient (β) and the Gaussian refinement intensity (σ) based on real-time optimization criteria such as progress ratio, population diversity, and fitness evolution. This is the key innovation in the adaptive machine learning-based parameter control method. While maintaining population diversity, the multi-leader guiding method with hierarchical weights (2.0, 1.5, and 1.0) provides a trustworthy search direction. When combined with progressive Gaussian refinement and reflective boundary treatment, this method effectively prevents premature convergence while allowing for detailed local search. The experimental results indicate that RIO-ML successfully employs intelligent adaptive learning to address the challenges of PV parameter estimation with outstanding accuracy, reliability, and robustness. The performance of the algorithm confirms its efficiency in estimating the photovoltaic parameters and its possible use in system modeling, performance analysis, and renewable energy optimization.

The RTC France benchmark I–V data, measured at standard test settings of 303.15 K and 1000 W/m², is used in this study to define and validate the PV parameter extraction problem. As a result, the experimental data used for parameter estimate implicitly reflects the irradiance effect. Therefore, the current formulation is meant to be used for benchmark evaluation in fixed operating conditions. Extending the framework to dynamically varying irradiance and temperature conditions represents an important direction for future work and would require explicit incorporation of irradiance-dependent photocurrent modeling and additional outdoor validation datasets.

## Data Availability

The datasets used and analyzed during the current study are included in the manuscript.

## Abbreviations

N: Number of agents (population size)
D: Number of dimensions (problem dimensionality)
$\:{T}_{max}$: Maximum number of iterations
t: Current iteration number
$\:{x}_{i}$: Position vector of agent i
$\:{x}_{ij}$: Position of agent i in dimension j
$\:{lb}_{j}$, $\:{ub}_{j}$: Lower and upper bounds for dimension j
f(x): Objective function value
$\:{pbest}_{i}$: Personal best position of agent i
L₁, L₂, L₃: Three best leader solutions
β: Social-reflective balance parameter [0.2, 0.9]
σ: Gaussian refinement standard deviation [0.001, 0.5]
a: Convergence parameter linearly decreasing from 2 to 0
$\:{p}_{refine}$: Refinement probability increasing from 0.3 to 1.0
$\:{A}_{k}$, $\:{C}_{k}$: Whale optimization coefficients for leader k
$\:{D}_{k}$: Distance to leader k
$\:{X}_{k}$: Candidate position based on leader k
G: Gaussian random variable ~ N(0, σ)
$\:{MLP}_{\beta\:}$, $\:{MLP}_{\sigma\:}$: Multi-Layer Perceptron regression models
SDM, DDM, TDM: Single, double and triple diode models

## Code availability

The code used to implement the proposed RIO-ML algorithm and perform the parameter estimation of photovoltaic models is available from the corresponding author on reasonable request.
